# Glioblastoma Segmentation: Comparison of Three Different Software Packages

**DOI:** 10.1371/journal.pone.0164891

**Published:** 2016-10-25

**Authors:** Even Hovig Fyllingen, Anne Line Stensjøen, Erik Magnus Berntsen, Ole Solheim, Ingerid Reinertsen

**Affiliations:** 1 Department of Neurosurgery, St. Olav’s University Hospital, Trondheim, Norway; 2 Department of Circulation and Medical Imaging, Faculty of Medicine, Norwegian University of Science and Technology, Trondheim, Norway; 3 Department of Radiology and Nuclear Medicine, St. Olav’s University Hospital, Trondheim, Norway; 4 Department of Neuroscience, Faculty of Medicine, Norwegian University of Science and Technology, Trondheim, Norway; 5 SINTEF, Technology and Society, Dept. Medical technology, Trondheim, Norway; Center for Neuroscience and Regenerative Medicine, UNITED STATES

## Abstract

To facilitate a more widespread use of volumetric tumor segmentation in clinical studies, there is an urgent need for reliable, user-friendly segmentation software. The aim of this study was therefore to compare three different software packages for semi-automatic brain tumor segmentation of glioblastoma; namely BrainVoyager^TM^ QX, ITK-Snap and 3D Slicer, and to make data available for future reference. Pre-operative, contrast enhanced T_1_-weighted 1.5 or 3 Tesla Magnetic Resonance Imaging (MRI) scans were obtained in 20 consecutive patients who underwent surgery for glioblastoma. MRI scans were segmented twice in each software package by two investigators. Intra-rater, inter-rater and between-software agreement was compared by using differences of means with 95% limits of agreement (LoA), Dice’s similarity coefficients (DSC) and Hausdorff distance (HD). Time expenditure of segmentations was measured using a stopwatch. Eighteen tumors were included in the analyses. Inter-rater agreement was highest for BrainVoyager with difference of means of 0.19 mL and 95% LoA from -2.42 mL to 2.81 mL. Between-software agreement and 95% LoA were very similar for the different software packages. Intra-rater, inter-rater and between-software DSC were ≥ 0.93 in all analyses. Time expenditure was approximately 41 min per segmentation in BrainVoyager, and 18 min per segmentation in both 3D Slicer and ITK-Snap. Our main findings were that there is a high agreement within and between the software packages in terms of small intra-rater, inter-rater and between-software differences of means and high Dice’s similarity coefficients. Time expenditure was highest for BrainVoyager, but all software packages were relatively time-consuming, which may limit usability in an everyday clinical setting.

## Introduction

Glioblastomas are diffusely infiltrating tumors, and the true tumor volume is larger and more diffuse than observed with any current imaging modality [1–4]. However, to study tumor growth dynamics, surgical results, or tumor response after oncological treatment, assessment of tumor volume is crucial [5]. By convention, tumor appearance in contrast enhanced T_1_-weighted magnetic resonance images (MRIs) defines the tumor volume in quantitative studies, but methods of assessment vary much between studies [6]. Manual volume segmentation of all T_1_ MRI slices remains the gold standard for volume measurement. However, this is very time-consuming, and some authors have questioned the accuracy of manual tumor delineation [7, 8]. Despite some exceptions [9–13], manual segmentation is not often used in larger studies. Some studies have instead used relatively simple geometric formulae based on post-contrast neuroimaging [14–16], and in many studies where progression-free survival is assessed, volume measures are based on crude assessments of tumor diameters (RECIST criteria, MacDonald Criteria, WHO, RANO criteria) [6, 17, 18]. With such diverging methodology, between-study comparisons are difficult.

In later years, several semi-automatic or fully automatic segmentation methods have been developed to save time and resources and to improve reliability of volume measurements [7, 19–21]. The simplest of these segmentation techniques is based on all voxels within a contrast-enhanced border, and have the potential of precisely estimating pre- and postoperative volumes. When combined with a well-developed graphical user interface, they can be implemented in an everyday clinical setting and in clinical studies. However, there are few validation studies or comparative studies of segmentation algorithms in clinical data sets. The nearly automatic methods generally rely on input from several MRI sequences, and generally require a high technical knowledge of the user [7, 20]. Semi-automatic methods more often employ only one type of MRI sequence, and could be easier to implement in a clinical research environment.

The aim of this study was to compare three different software packages for semi-automatic segmentation of glioblastoma; namely BrainVoyager^TM^ QX, ITK-Snap and 3D Slicer. We assessed intra- and inter-rater agreement in pre-operative tumor volumes with between-software comparisons. Time expenditure was recorded as a measure of usability in everyday clinical practice. By publishing results and image data, we also seek to establish a possible reference database, which could be utilized to compare future segmentation methods.

## Method and Materials

### Study design and participants

**Fig 1:**
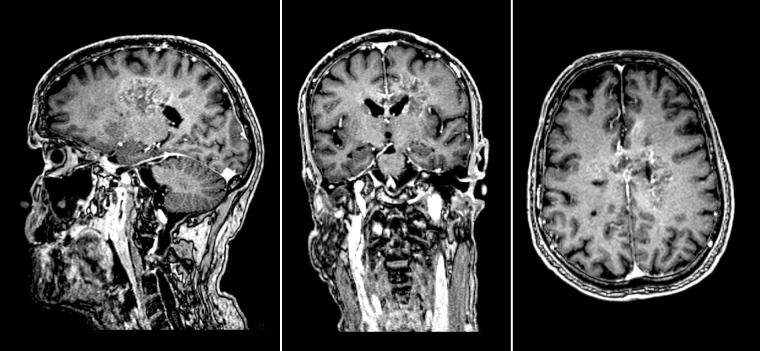
Example of a case excluded due to very diffuse contrast enhancing properties.

St. Olav’s University Hospital has a region-based referral practice where all brain tumors in the region are referred to our Neurosurgical department. A sample of 20 consecutive patients who had undergone primary surgery for glioblastoma was included. The exclusion criteria were: earlier brain tumor surgery or radiotherapy, non-contrast-enhancing lesions, gliomatosis cerebri, unavailable or unsuitable preoperative MRI scans (no contrast given, unacceptable motion artifacts). Two tumors were later excluded due to low and/or diffuse contrast enhancing properties in two of the MRI scans making reliable segmentation nearly impossible, as judged by a neuroradiologist (E.M.B). Therefore, only 18 were included in the analyses. An example of an excluded tumor is presented as Fig 1. The study was approved by the Regional Ethics Committee (Central) as part of a larger project (reference 2013–1348), with waiver of informed consent for retrospective review of MRI images obtained as part of clinical routine.

### Data acquisition

Pre-operative contrast enhanced 3D T_1_ MPRAGE (Magnetization-Prepared Rapid Acquisition Gradient-Echo) images acquired at 1.5 or 3 Tesla were retrospectively obtained from the St. Olav’s University Hospital’s radiology database. MRI scans had been performed on three different scanner systems; exact scan parameters for each case are presented in S1 Table. All scans had been obtained using a gadolinium based contrast agent (Dotarem; Bayer Schering Pharma, Leverkusen, Germany and Omniscan; GE Healthcare, Little Chalfont, Buckinghamshire, United Kingdom). Scans were de-identified by one of the authors (E.M.B) and segmented independently by two investigators (A.L.S, E.H.F) using three different software packages: BrainVoyager^TM^ QX version 1.2 (Brain Innovation B.V., the Netherlands, available at www.brainvoyager.com) [22], 3D Slicer version 4.3.1 (www.slicer.org) [19] and ITK-Snap version 3.0.0 (www.itksnap.org) [21]. The choice of software solutions was guided by the following criteria: (1) BrainVoyager was included because this had been the software previously used for brain tumor segmentation in our group, and it was thus natural to compare other software with this. (2) The software should offer semi-automatic segmentation from a single MRI sequence (contrast enhanced T_1_ in this case). (3) The software should also have a graphical user interface that could be used without specific technical knowledge. (4) Preferably, the software should be open source and free of charge. ITK-Snap and 3D Slicer are both free and open-source, while BrainVoyager requires a license.

Tumor volume was defined as the pathological contrast enhancement plus the necrotic tissue within the contrast enhancing borders. Non-enhancing bulk tumor, indicating a transformed lower grade glioma, was not seen in any of the included patients. At the time of segmentation, A.L.S was a medical student trained in segmentation by a neuroradiologist (E.M.B), and E.H.F was a resident neurosurgeon. As part of a different project, A.L.S had segmented 106 GBMs in BrainVoyager before the start of this study [13]. Each tumor was segmented two times in each software package by each of the two investigators with a minimum of 14 days between segmentations of the same tumor. Thus, tumors were segmented six times per investigator to a total of twelve segmentations per tumor. After segmentation, E.M.B performed a visual control of the first segmentation round in BrainVoyager by both A.L.S and E.H.F as quality control. The inspection revealed that all tumors were segmented after the contrast-enhancing border, no obvious non-tumor tissue was included, and all segmentations were considered to delineate the tumor very well. No segmentations were evaluated as in need of correction after control.

### Segmentation procedures

In BrainVoyager, the authors first selected a 3D volume with a box tool that covered the entire tumor. Subsequently, a range algorithm was applied, that selected all voxels with intensity above a defined threshold within this volume. Contrast enhanced voxels not representing tumor (meninges, blood vessels, plexus choroideus) were manually deselected from each slice with an in-software tool. The resulting segmented volume comprised the contrast enhancing tumor tissue. The procedure was repeated for the voxels with intensity below the above-defined threshold within the contrast enhancing limits to select necrotic parts of the tumor. The volume was then calculated from the voxel size and the number of voxels segmented.

In 3D Slicer, a competitive region-based segmentation module called “GrowCut” was used. The workflow has previously been described by others [19]. In short, very rough inner and outer tumor borders were selected in three orthogonal planes before applying the GrowCut function. The software calculated the rough borders of the tumor and presented this as colored areas on the images. These borders were refined using the functions “Dilate” and “Erode” with 8 pixel neighbors and a manual segmentation tool, before calculating the volume of the tumor.

ITK-Snap is based on a combination of geodesic active contours and region competition in order to create an evolving estimate of tumor borders [21]. Using the Active Contour Segmentation Mode with region competition, different growing points in the contrast-enhancing tumor parts were selected to “grow” the algorithm. The evolving estimate was graphically displayed as a color label. We stopped the algorithm when there was no further algorithm growth for approximately 5 seconds, or when the estimated tumor borders leaked outside the contrast-enhancing tumor. The central non-enhancing tumor tissue often had to be manually added to the segmentation result.

**Fig 2:**
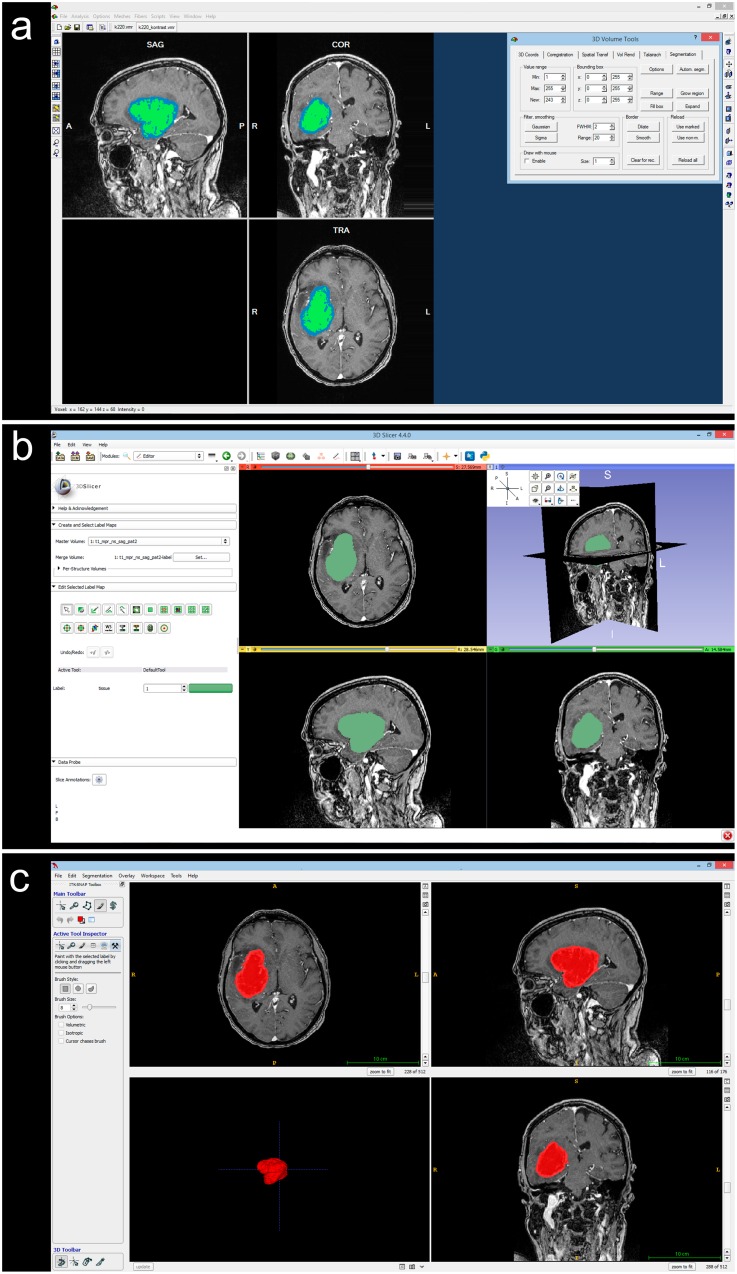
Examples of user-interface for segmentation in each software package. a BrainVoyager, b 3D Slicer, c ITK-Snap.

More comprehensive segmentation workflows with illustration images can be found in the S1 Appendix. Examples of tumor segmentations in each software package with colored segmentation labels are presented as Fig 2. Time expenditure for segmentation was measured for all segmentations by A.L.S using a stopwatch.

### Statistical considerations

Intra-rater agreement was calculated as difference of tumor volume means with 95% limits of agreement (LoA) between the first and second segmentations for each of the investigators in each software package, as described by Bland and Altman [23]. Inter-rater agreement was calculated in the same manner using difference of tumor volume means with 95% LoA between investigators for the second segmentations in each software package. A combined mean tumor volume for each software package was calculated based on the second segmentations of both investigators. Agreement between software packages was calculated by the difference of these combined tumor volume means with 95% LoA. We used the second segmentations as basis for inter-rater and between-software agreement. Bland-Altman plots [24] with 95% LoA were created from the above calculated values.

To compare our results to the results obtained for automatic glioma segmentation in the BRATS benchmark, a spatial overlap index was calculated [20]. Specifically, intra-rater Dice’s similarity coefficients (DSC) were calculated for both investigators [25]. Inter-rater and between-software DSC and Hausdorff distances (HD) were calculated, again using the second segmentation for each rater [26]. Between-software DSC and HD were calculated separately for each of the investigators. DSC was in this study used to measure spatial overlap between tumor segmentations with 0 representing no overlap and 1 representing perfect overlap. DSC is a widely used measure of segmentation overlap, and is relatively insensitive to outliers. A DSC of > 0.700 is regarded as good overlap [27]. HD was used as a measure of the longest distance between two voxels on the outer borders of two segmentations for a random set of voxels. HD is sensitive for variations in segmentation borders and small volume outliers.

In order to explore the magnitude of differences in volume between segmentations relative to the tumor volume, we calculated the difference in volume between segmentations as a percent of tumor volume. Differences in time expenditure between segmentation rounds and between software packages were analyzed using paired samples *t*-test. Simple plots of mean tumor volumes with 95% confidence intervals were created in order to investigate any trend towards increasing or decreasing segmentation volumes with repeated segmentations as a sign of a possible learning curve in image interpretation.

3D Slicer version 3.2.1 with the DiceComputation module was used to calculate DSC, while the ModelToModelDistance module was used to calculate HD (both downloaded through the 3D Slicer built-in extension manager). IBM SPSS Statistics version 22.0.0.2 64-bit for OS X was used for all other analyses.

## Results

**Fig 3:**
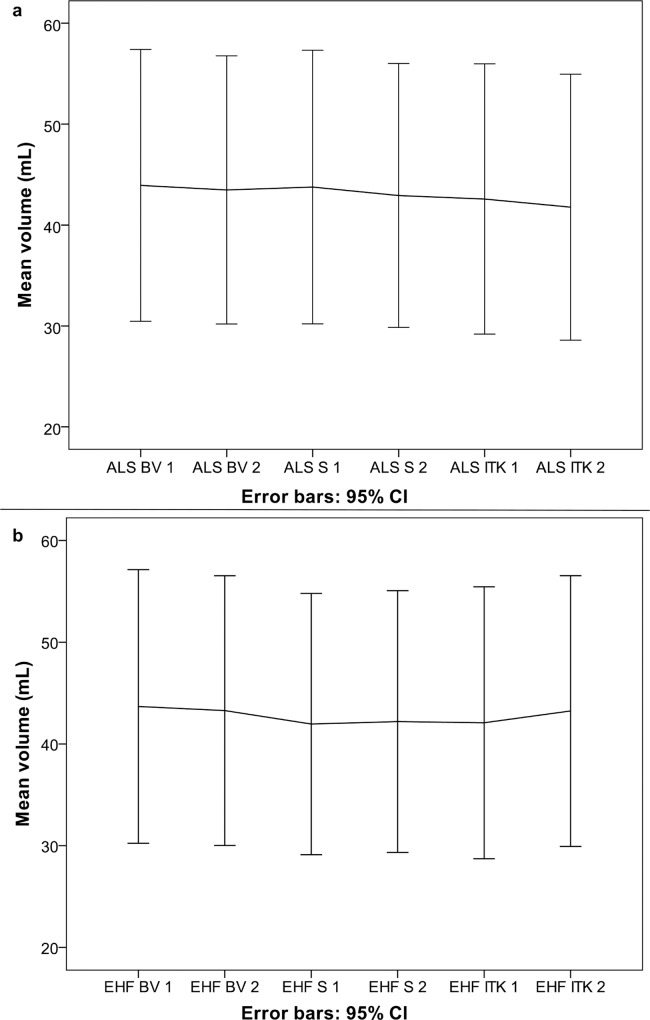
Mean volumes with 95% CI for the two raters in the different software solutions. a Segmentations by A.L.S, b segmentations by E.H.F.

**Fig 4:**
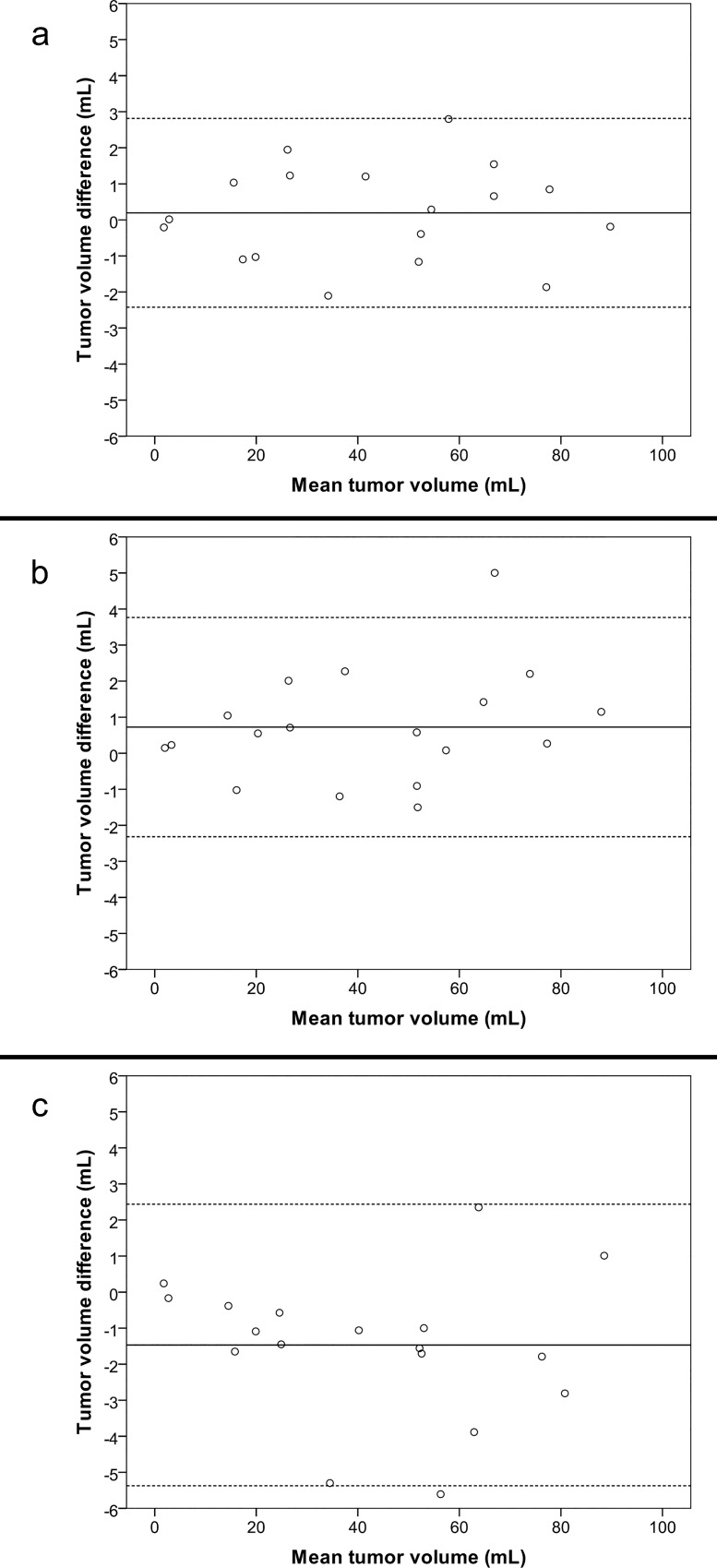
Inter-rater agreement. Difference in tumor volumes for second segmentations between investigators using each software plotted against mean tumor volume of each segmentation (inter-rater agreement). Whole line represents mean difference, stapled lines represent 95% limits of agreement. a BrainVoyager, b 3D Slicer, c ITK-Snap

**Fig 5:**
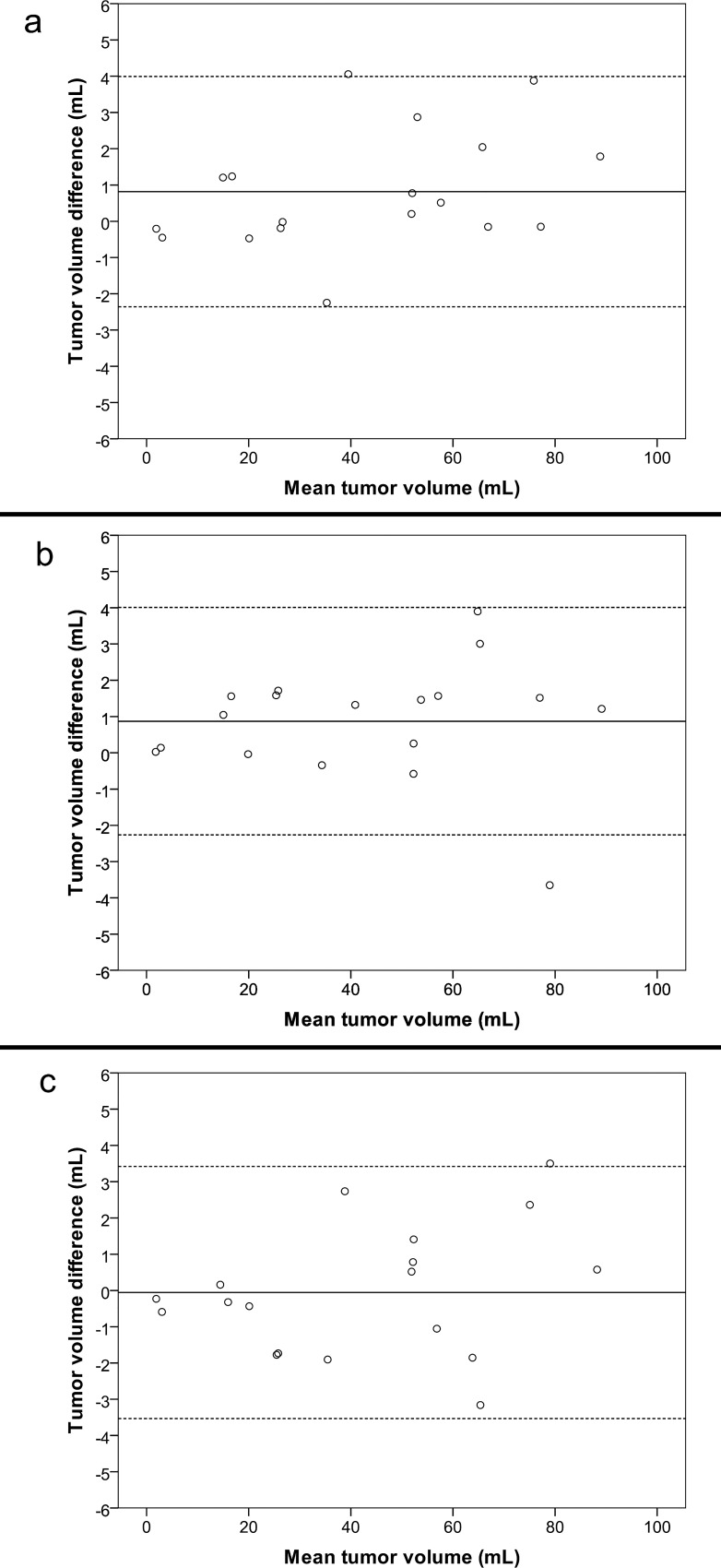
Between-software agreement. Difference in tumor volumes for combined mean of second segmentations for investigators between software packages plotted against mean tumor volume of each segmentation. Whole line represents mean difference, stapled lines represent 95% limits of agreement. a BrainVoyager and 3D Slicer, b BrainVoyager and ITK-Snap, c ITK-Snap and 3D Slicer

There was no systematic increase or decrease in mean segmentation volume with repeated segmentations for any of the two investigators (Fig 3). Descriptive statistics of tumor volumes are presented in Table 1. The intra-rater agreement, inter-rater agreement and between-software agreement are presented in Table 2. Inter-rater agreement was highest for BrainVoyager with a difference of means of 0.19 mL and 95% LoA from -2.42 mL to 2.81 mL. Analyses of between-software agreement resulted in very similar differences of means and 95% LoA to the inter-rater analyses. Bland-Altman plots for inter-rater and between-software agreement with 95% LoA are presented as Figs 4 and 5.

**Table 1:** Descriptive statistics for tumor volume segmentations in mL for each investigator (N = 18).

	A.L.S	E.H.F
	Mean (standard deviation, range)	Mean (standard deviation, range)
***BrainVoyager***		
***First segmentation***	43.9 (27.1, 1.7 to 91.7)	43.7 (27.0, 2.0 to 89.7)
***Second segmentation***	43.5 (26.7, 1.7 to 89.6)	43.3 (26.6, 1.9 to 89.8)
***3D Slicer***		
***First segmentation***	43.8 (27.2, 1.9 to 91.1)	42.0 (25.8, 1.8 to 87.4)
***Second segmentation***	42.9 (26.3, 2.1 to 88.5)	42.2 (25.9, 1.9 to 87.4)
***ITK-Snap***		
***First segmentation***	42.6 (26.9, 1.9 to 89.2)	42.1 (26.9, 1.8 to 87.2)
***Second segmentation***	41.8 (26.5, 1.9 to 89.0)	43.2 (26.8, 1.7 to 88.0)

**Table 2:** Intra-rater, Inter-rater and Between-software agreement reported as difference of means (mL) with 95% LoA.

***Intra-rater agreement***	*A*.*L*.*S*	*E*.*H*.*F*
	*Difference of means (95% LoA)*	*Difference of means (95% LoA)*
BrainVoyager	0.45 (-1.34 to 2.25)	0.40 (-1.70 to 2.50)
3D Slicer	0.84 (-1.93 to 3.61)	-0.23 (-1.70 to 1.24)
ITK-Snap	0.80 (-2.69 to 4.30)	-1.16 (-5.41 to 3.09)
***Inter-rater agreement***	*Difference of means (95% LoA)*	
BrainVoyager	0.19 (-2.42 to 2.81)	
3D Slicer	0.72 (-2.32 to 3.77)	
ITK-Snap	-1.47 (-5.37 to 2.44)	
***Between-software agreement***		
BrainVoyager vs. 3D Slicer	0.82 (-2.36 to 3.99)	
BrainVoyager vs. ITK-Snap	0.87 (-2.26 to 4.01)	
ITK-Snap vs. 3D Slicer	-0.06 (-3.53 to 3.42)	

Difference of tumor volume means in mL with 95% LoA (limits of agreement) between first and second segmentations for each investigator (A.L.S, E.H.F, intra-rater agreement), between investigators using second segmentations (inter-rater agreement), and between software packages using the combined mean of the second segmentations (between-software agreement) (N = 18)

Inter-rater mean difference in volume was 3.7% (range 0.2% to 11.6%) for BrainVoyager, 3.9% (range 0.1% to 7.6%) for 3D Slicer and 5.5% (range 1.1% to 15.4%) for ITK-Snap (S3 Table). Between-software mean difference in volume was 4.4% (range 0.1% to 14.7%) between BrainVoyager and 3D Slicer, 3.7% (range 0.2% to 9.4%) between BrainVoyager and ITK-Snap and 4.8% (range 0.7% to 19.7%) between 3D Slicer and ITK-Snap. The largest differences in percent were with the smallest tumors, where small variations in segmentation volumes result in large differences in percent.

Dice’s similarity coefficients (DSC) with Hausdorff distance (HD) between investigators and between software packages are presented in Tables 3 and 4. All in all, there were high DSC for all analyses, with all mean DSC for intra-rater, inter-rater and between-software segmentations ≥ 0.93 (S4 Table). We identified two cases that skewed the HD; in two cases of BrainVoyager segmentations by E.H.F, there were a few voxels lying far away from the tumor resulting in HD as high as 74.3 mm. As such, HD are presented as median values. Because the HD is sampled in a random set of voxels and not all voxels, maximum HD were only affected in between-software analyses and not inter-rater analyses. In two segmentations in 3D Slicer by E.H.F, a missing satellite tumor was discovered when comparing volume models between segmentations. The satellite tumors were then segmented and included in the analyses, as the focus of this study was agreement between segmentation algorithms and not a test of radiological diagnostics. This only resulted in minimal changes in mean tumor volume (≤ 0.01 mL), differences of means (change in difference of means ≤ 0.01 mL and change in 95% LoA ≤ 0.04 mL for all affected analyses) or DSC (≤ 0.01 for all affected analyses), but the maximum HD was reduced from 16.7 mm to 8.0 mm when comparing second segmentations in 3D Slicer between investigators, and from 16.3 mm to 9.2 mm for E.H.F segmentations in ITK-Snap vs. 3D Slicer segmentations. All numbers reported in this paper and the supplementary information are after these corrections.

**Table 3:** Inter-rater Dice’s similarity coefficients (DSC) and Hausdorff distance in millimeters (HD) (N = 18).

	*Mean DSC (range)*	*Median HD (range)*
***BrainVoyager***	0.96 (0.89 to 1.00)	3.9 (2.3 to 7.1)
***3D Slicer***	0.95 (0.90 to 0.99)	4.6 (2.2 to 8.0)
***ITK-Snap***	0.95 (0.89 to 0.99)	4.8 (2.4 to 12.9)

**Table 4:** Between software Dice’s similarity coefficients (DSC) and Hausdorff distance in millimeters (HD), for each rater (N = 18).

	A.L.S		E.H.F	
	*Mean DSC (range)*	*Median HD (range)*	*Mean DSC (range)*	*Median HD (range)*
***BrainVoyager vs*. *3D Slicer***	0.93 (0.82 to 0.98)	4.9 (2.2 to 7.8)	0.93 (0.84 to 0.98)	6.4 (2.3 to 74.3)
***BrainVoyager vs*. *ITK-Snap***	0.94 (0.82 to 0.99)	4.3 (2.4 to 8.5)	0.94 (0.83 to 0.98)	5.3 (2.8 to 74.0)
***ITK-Snap vs*. *3D Slicer***	0.94 (0.84 to 0.98)	5.8 (2.0 to 12.9)	0.94 (0.88 to 0.99)	5.3 (2.2 to 9.2)

The mean time expenditure for the second segmentation by A.L.S for BrainVoyager, 3D Slicer and ITK-Snap was 41 min (range 13 min to 1 h 19 min), 18 min (range 5 min to 30 min) and 18 min (range 6 min to 39 min), respectively (S6 Table). The difference in time expenditure was statistically significant between BrainVoyager and 3D Slicer (P < 0.001), and between BrainVoyager and ITK-Snap (P < 0.001), but not between 3D Slicer and ITK-Snap (P = 0.887). There was a significant decrease in mean time expenditure between first and second segmentations by A.L.S in BrainVoyager and 3D Slicer by 7 min 33 sec (95% CI 1 min 07 sec to 14 min 0 sec, P = 0.024) and 4 min 49 sec (95% CI 1 min 21 sec to 8 min 16 sec, P = 0.009), respectively. No such effect was found for ITK-Snap (mean difference 6 sec, P = 0.927). Running the GrowCut function in 3D Slicer took from 21 sec to 6 min 5 sec using a midrange portable computer (Intel Core i5-3317U 1,7 GHz processor and 4 GB RAM).

## Discussion

This is the first study to explore agreement between these three semi-automatic brain tumor segmentation software packages for segmentation of glioblastoma. There was high agreement within and between software packages in terms of small intra-rater, inter-rater and between-software differences of means and high DSC. BrainVoyager, the least automatic and by far most time-consuming segmentation method, did not perform better than the more automatic methods in terms of agreement.

Differences of means and 95% LoA between software packages are comparable to the intra- and inter-rater differences of means and 95% LoA, indicating similar performance of the three software packages. Further, all the mean DSC were ≥ 0.93, indicating very good spatial overlap between segmentations. Results from 20 state-of-the-art multimodal automatic segmentation algorithms were published by Menze et al. in 2015 [20]. In that study, T_1_-weighted, T_2_-weighted and FLAIR images from high grade gliomas were used to segment different tumor compartments, which then were used for validation of automatic segmentation results. The regions used for validation were the whole tumor including edema, “tumor core” and “enhancing tumor core”, of which “tumor core” corresponds best to our definition of the tumor volume. The manual segmentations of the tumor core of high-grade gliomas in the study by Menze *et al*. had a mean inter-rater DSC of 0.93. When choosing the best segmentation of each tumor from all the automatic algorithms, they obtained a DSC of 0.82 for tumor core in high grade gliomas. This would be the theoretical upper limit of segmentation overlap of the automatic algorithms they tested [20]. Compared to their manual segmentations, we achieved higher mean inter-rater DSC for all software packages. This is in line with recent studies showing better reproducibility and robustness with semi-automatic segmentation algorithms compared to manual segmentation in primary lung tumors and uterine fibroids using CT scans and MRI scans, respectively [8, 28]. High intra- and inter-rater agreement ensures reproducibility and enables comparisons across studies, and may be more important than agreement with manual segmentation, the current “gold standard”.

Using BrainVoyager, mean segmented volumes were slightly larger than for the other software packages. The investigators experienced that they had a tendency of slightly increasing the tumor edges when the software applied the color mask because one could then easier visualize the differences in contrast outside of the color mask. When removing the color mask, it was often seen that the mask was slightly too large. As the human eye over-emphasizes contrast, tumor volume may by this workflow be over-estimated and result in measurement bias.

When exploring mean difference in volume as a percent of tumor volume, we found that the largest differences in percent were in the smallest tumors. This reflects that relatively small variations in segmentation volume will tend to result in larger differences in percent for small tumors. However, when comparing this with Figs 4 and 5, small volume segmentations still have smaller tumor volume differences than larger tumors in mL.

Compared to the study by Egger *et al*. using 3D Slicer for segmentation of brain tumors, we had higher DSC, but also higher HD [19]. Higher HD was especially evident in a few of the segmentations using BrainVoyager, where voxels had been erroneously included in the segmentations because they had not been manually removed, as the workflow in this software requires. This has little impact on DSC and tumor volume estimations, but underlines the need for segmentation software that requires as little manual adjustment as possible in order to minimize sources of error.

Despite optimal treatment glioblastoma almost invariably progresses, and the evaluation of disease progression and response to therapy is based on radiological assessment. Volumetric tumor segmentations are likely to be more sensitive [29, 30] than crude measures used in current guidelines. Today, so called “progression free” survival is often defined as less than a 20% increase in the sum of diameters of a tumor [17]. In an ellipsoid shaped lesion this equals approximately a doubling of the volume. Thus, smaller treatment effects can potentially be missed and statistical power is lower than with use of more sensitive measures [5]. Also, glioblastomas, and especially tumor remnants, are usually not ellipsoid shaped, hampering volume measures based on crude diameters. Segmentation can also be of clinical use. Pre-operative 3D segmentation can improve visualization for the surgeon both during planning and intra-operatively when using neuro-navigation. Also, volume segmentation may be useful for planning radiotherapy or evaluating treatment effects in individual patients.

For everyday clinical work, time expenditure was higher than desired for all software packages. This especially applies for BrainVoyager, which was considerably more time consuming than the two other software packages. Running the GrowCut algorithm in 3D Slicer took up to about 6 minutes, and total segmentation time expenditure may therefore be somewhat reduced on a more powerful computer. For segmentation to become part of everyday practice, it is imperative that state-of-the-art algorithms are implemented in user-friendly graphical user-interfaces that are accessible to clinicians and engineers alike.

A possible limitation of the present study was that none of the investigators who performed the segmentations were neuroradiologists, despite considerable experience with assessment of brain tumors. Because tumor margins were defined as the edges of contrast enhancement on MRI scans, tumor borders were readily visible in most cases. Possible pitfalls are contrast enhancement in tumor-near blood vessels or vessels entering the tumors, in addition to other contrast enhancing tissue such as the choroidal plexus. However, as the tumor could be visualized in all three orthogonal planes in all programs, this is not likely to be a major source of error. Satellite tumors represent a different kind of pitfall.

In this study we defined the tumor volume as the contrast enhancing compartment together with the central necrosis in contrast enhanced T_1_-weighted images. This is a commonly used glioblastoma tumor volume definition, but it could be seen as a limitation to our study that we lack information about segmentation of tumor in T_2_/FLAIR images. The contrast enhancing compartment, with or without the central necrosis, is currently being used for measuring extent of tumor resection, preoperative tumor growth, and progression of tumors after treatment, as suggested in the RANO criteria [6, 31, 32]. For primary glioblastomas, the most relevant additional tumor compartment to segment would be the edema surrounding the tumor, as seen on T_2_ or FLAIR images, which is known to encompass varying degrees of infiltrating tumor cells. However, as stated in the RANO-criteria, several other disease processes, such as infarctions, might resemble the appearance of tumor edema in these images [6]. In addition, it is not possible to differentiate edema due to tumor infiltration from reactive edema based on T_2_/FLAIR images alone [5, 33]. From a technical point of view, there exists many automatic algorithms which produce segmentations of several different tumor compartments. However, in the BRATS challenge, no one algorithm produced optimal results for all the three included tumor regions [20]. Of the software solutions we tested, none could deliver concurrent multimodal semi-automatic segmentation. However, it would be possible to segment T_2_/FLAIR and T_1_-images separately. As shown by Menze et al., segmentation of the whole tumor including edema is generally easier than to segment contrast-enhancing tumor [20]. If a standard definition of tumor on T_2_/FLAIR images was chosen, the software tested in our study could be used, but the time usage would be an even stronger practical limitation.

The MRI scans were obtained from different MRI scanners with varying field strengths (both 1.5 T and 3 T). As such, the scan parameters obtained were not standardized. By analyzing differences of means and DSC this is unlikely to contribute to bias, though precision of the volume estimates may vary with quality of images. However, one may argue that this more closely resembles the day-to-day workflow with different scanners and scan parameters.

## Conclusions

In this study, we have found the software packages BrainVoyager, 3D Slicer and ITK-Snap to have similar performance in terms of high intra-rater, inter-rater and between-software volume agreement and spatial overlap for segmentation of GBM. Time expenditure was significantly longer in BrainVoyager than in the two other software packages, but both time expenditure and smoothness of workflow could be better for all three software packages.

## Supporting Information

S1 Appendix Comprehensive segmentation workflows in each software. (DOCX)

S1 Table Overview of scan parameters for all MRI scans (DOC)

S2 Table Segmented volumes for all tumors in all segmentation rounds (XLSX)

S3 Table Intra-rater, inter-rater and between software relative differences for all segmentations (XLSX)

S4 Table Intra-rater, inter-rater and between software DSC values for all segmentations (XLSX)

S5 Table Inter-rater and between-software HD values for all segmentations (XLSX)

S6 Table Time usage by ALS for all segmentations (XLSX)

## References

[1] Scherer H. The forms of growth in gliomas and their practical significance. Brain. 1940;63(1):1–35.

[2] Matsukado Y, Maccarty CS, Kernohan JW. The growth of glioblastoma multiforme (astrocytomas, grades 3 and 4) in neurosurgical practice. J Neurosurg. 1961;18:636–44. 10.3171/jns.1961.18.5.0636 13768222

[3] Kelly PJ, Daumas-Duport C, Kispert DB, Kall BA, Scheithauer BW, Illig JJ. Imaging-based stereotaxic serial biopsies in untreated intracranial glial neoplasms. J Neurosurg. 1987;66(6):865–74. 10.3171/jns.1987.66.6.0865 3033172

[4] Tovi M, Hartman M, Lilja A, Ericsson A. MR imaging in cerebral gliomas. Tissue component analysis in correlation with histopathology of whole-brain specimens. Acta Radiol. 1994;35(5):495–505. 8086262

[5] Sorensen AG, Batchelor TT, Wen P, Zhang WT, Jain RK. Response criteria for glioma. Nature Clinical Practice Oncology. 2008;5(11):634–44. 10.1038/Ncponc1204 18711427 PMC4795821

[6] Wen PY, Macdonald DR, Reardon DA, Cloughesy TF, Sorensen AG, Galanis E, et al Updated Response Assessment Criteria for High-Grade Gliomas: Response Assessment in Neuro-Oncology Working Group. J Clin Oncol. 2010;28(11):1963–72. 10.1200/jco.2009.26.3541 20231676

[7] Gordillo N, Montseny E, Sobrevilla P. State of the art survey on MRI brain tumor segmentation. Magn Reson Imaging. 2013;31(8):1426–38. 10.1016/j.mri.2013.05.002 23790354

[8] Heye T, Merkle EM, Reiner CS, Davenport MS, Horvath JJ, Feuerlein S, et al Reproducibility of dynamic contrast-enhanced MR imaging. Part II. Comparison of intra- and interobserver variability with manual region of interest placement versus semiautomatic lesion segmentation and histogram analysis. Radiology. 2013;266(3):812–21. 10.1148/radiol.12120255 23220891

[9] Duong DH, Rostomily RC, Haynor DR, Keles GE, Berger MS. Measurement of tumor resection volumes from computerized images. Technical note. J Neurosurg. 1992;77(1):151–4. 10.3171/jns.1992.77.1.0151 1607959

[10] Shi WM, Wildrick DM, Sawaya R. Volumetric measurement of brain tumors from MR imaging. J Neurooncol. 1998;37(1):87–93. 9525843 10.1023/a:1005944724470

[11] Kuhnt D, Becker A, Ganslandt O, Bauer M, Buchfelder M, Nimsky C. Correlation of the extent of tumor volume resection and patient survival in surgery of glioblastoma multiforme with high-field intraoperative MRI guidance. Neuro Oncol. 2011;13(12):1339–48. 10.1093/neuonc/nor133 21914639 PMC3223093

[12] Sanai N, Polley MY, McDermott MW, Parsa AT, Berger MS. An extent of resection threshold for newly diagnosed glioblastomas. J Neurosurg. 2011;115(1):3–8. 10.3171/2011.2.jns10998 21417701

[13] Stensjøen AL, Solheim O, Kvistad KA, Håberg AK, Salvesen Ø, Berntsen EM. Growth dynamics of untreated glioblastomas in vivo. Neuro Oncol. 2015;17(10):1402–11. 10.1093/neuonc/nov029 25758748 PMC4578579

[14] Solheim O, Selbekk T, Jakola AS, Unsgard G. Ultrasound-guided operations in unselected high-grade gliomas—overall results, impact of image quality and patient selection. Acta Neurochir (Wien). 2010;152(11):1873–86. 10.1007/s00701-010-0731-5 20652608

[15] Stummer W, Pichlmeier U, Meinel T, Wiestler OD, Zanella F, Reulen HJ, et al Fluorescence-guided surgery with 5-aminolevulinic acid for resection of malignant glioma: a randomised controlled multicentre phase III trial. Lancet Oncol. 2006;7(5):392–401. 10.1016/S1470-2045(06)70665-9 16648043

[16] Zhang Z, Jiang H, Chen X, Bai J, Cui Y, Ren X, et al Identifying the survival subtypes of glioblastoma by quantitative volumetric analysis of MRI. J Neurooncol. 2014;119(1):207–14. 10.1007/s11060-014-1478-2 24828264

[17] Eisenhauer EA, Therasse P, Bogaerts J, Schwartz LH, Sargent D, Ford R, et al New response evaluation criteria in solid tumours: revised RECIST guideline (version 1.1). Eur J Cancer. 2009;45(2):228–47. 10.1016/j.ejca.2008.10.026 19097774

[18] Macdonald DR, Cascino TL, Schold SC, Cairncross JG. Response criteria for phase II studies of supratentorial malignant glioma. J Clin Oncol. 1990;8(7):1277–80. 2358840 10.1200/JCO.1990.8.7.1277

[19] Egger J, Kapur T, Fedorov A, Pieper S, Miller JV, Veeraraghavan H, et al GBM volumetry using the 3D slicer medical image computing platform. Sci Rep. 2013;3 10.1038/srep01364 23455483 PMC3586703

[20] Menze BH, Jakab A, Bauer S, Kalpathy-Cramer J, Farahani K, Kirby J, et al The Multimodal Brain Tumor Image Segmentation Benchmark (BRATS). IEEE Trans Med Imaging. 2015;34(10):1993–2024. 10.1109/TMI.2014.2377694 25494501 PMC4833122

[21] Yushkevich PA, Piven J, Hazlett HC, Smith RG, Ho S, Gee JC, et al User-guided 3D active contour segmentation of anatomical structures: significantly improved efficiency and reliability. Neuroimage. 2006;31(3):1116–28. 10.1016/j.neuroimage.2006.01.015 16545965

[22] Goebel R, Esposito F, Formisano E. Analysis of functional image analysis contest (FIAC) data with brainvoyager QX: From single-subject to cortically aligned group general linear model analysis and self-organizing group independent component analysis. Hum Brain Mapp. 2006;27(5):392–401. 10.1002/hbm.20249 16596654 PMC6871277

[23] Bland JM, Altman DG. Statistical methods for assessing agreement between two methods of clinical measurement. Lancet. 1986;1(8476):307–10. 2868172

[24] Bland JM, Altman DG. Measuring agreement in method comparison studies. Stat Methods Med Res. 1999;8(2):135–60. 10501650 10.1177/096228029900800204

[25] Dice LR. Measures of the Amount of Ecologic Association between Species. Ecology. 1945;26(3):297–302. 10.2307/1932409

[26] Hausdorff F. Grundzüge der Mengenlehre. Nachdruck: New York: Chelsea Publ; 1949.

[27] Zijdenbos AP, Dawant BM, Margolin RA, Palmer AC. Morphometric analysis of white matter lesions in MR images: method and validation. IEEE Trans Med Imaging. 1994;13(4):716–24. 10.1109/42.363096 18218550

[28] Parmar C, Rios Velazquez E, Leijenaar R, Jermoumi M, Carvalho S, Mak RH, et al Robust Radiomics Feature Quantification Using Semiautomatic Volumetric Segmentation. PLoS ONE. 2014;9(7):e102107 10.1371/journal.pone.0102107 25025374 PMC4098900

[29] Reuter M, Gerstner ER, Rapalino O, Batchelor TT, Rosen B, Fischl B. Impact of MRI head placement on glioma response assessment. J Neurooncol. 2014;118(1):123–9. 10.1007/s11060-014-1403-8 24566765 PMC4026260

[30] Clarke LP, Velthuizen RP, Clark M, Gaviria J, Hall L, Goldgof D, et al MRI Measurement of Brain Tumor Response: Comparison of Visual Metric and Automatic Segmentation. Magn Reson Imaging. 1998;16(3):271–9. 10.1016/S0730-725X(97)00302-0 9621968

[31] Lacroix M, Abi-Said D, Fourney DR, Gokaslan ZL, Shi W, DeMonte F, et al A multivariate analysis of 416 patients with glioblastoma multiforme: prognosis, extent of resection, and survival. J Neurosurg. 2001;95(2):190–8. 10.3171/jns.2001.95.2.0190 11780887

[32] Ellingson BM, Nguyen HN, Lai A, Nechifor RE, Zaw O, Pope WB, et al Contrast-enhancing tumor growth dynamics of preoperative, treatment-naive human glioblastoma. Cancer. 2016;122(11):1718–27. 10.1002/cncr.29957 26998740

[33] Barajas RF Jr., Phillips JJ, Parvataneni R, Molinaro A, Essock-Burns E, Bourne G, et al Regional variation in histopathologic features of tumor specimens from treatment-naive glioblastoma correlates with anatomic and physiologic MR Imaging. Neuro Oncol. 2012;14(7):942–54. 10.1093/neuonc/nos128 22711606 PMC3379808

