# Supplementary material for: Glioblastoma Segmentation: Comparison of Three Different Software Packages
Source: PLoS One. 2016 Oct 25;11(10):e0164891. doi: 10.1371/journal.pone.0164891 (PMC5079567; doi:10.1371/journal.pone.0164891)

# S1 Appendix comprehensive segmentation workflows

## Segmentation workflow in BrainVoyager

The following workflow was used:

1. Images were loaded as dicom files into the software, which then transformed them to a .vmr-file.
2. The operator defined a 3D volume of interest enclosing the tumor (a), and by using a range-algorithm, voxels with intensity within an arbitrary range (b) were marked.


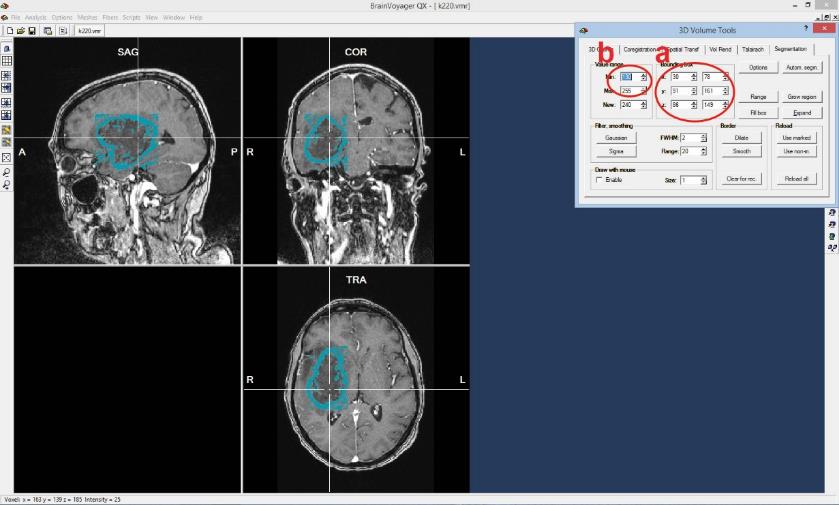


1. The marked voxels were then saved separately as a mask. The intensity-threshold for the mask was determined visually by including all voxels of the contrast enhancement.
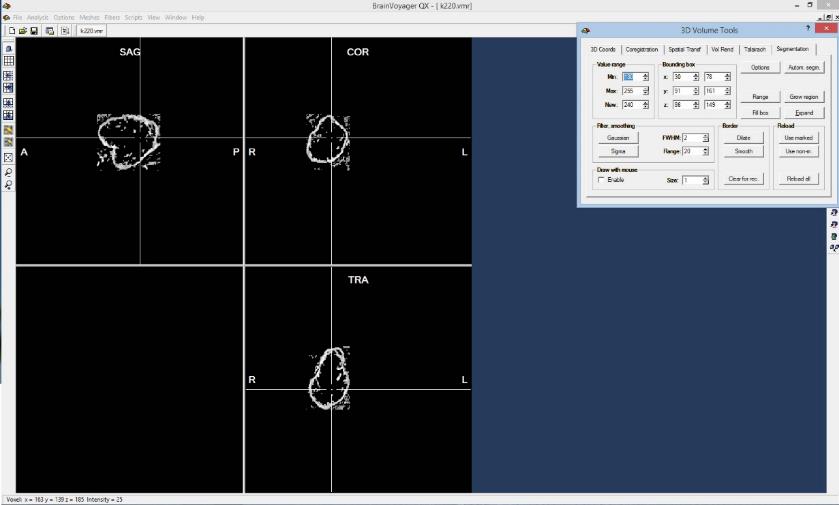

2. The mask was corrected by the operator using draw- and erase-tools, so that voxels that did not contain contrast enhancing tumor tissue were excluded from the mask.


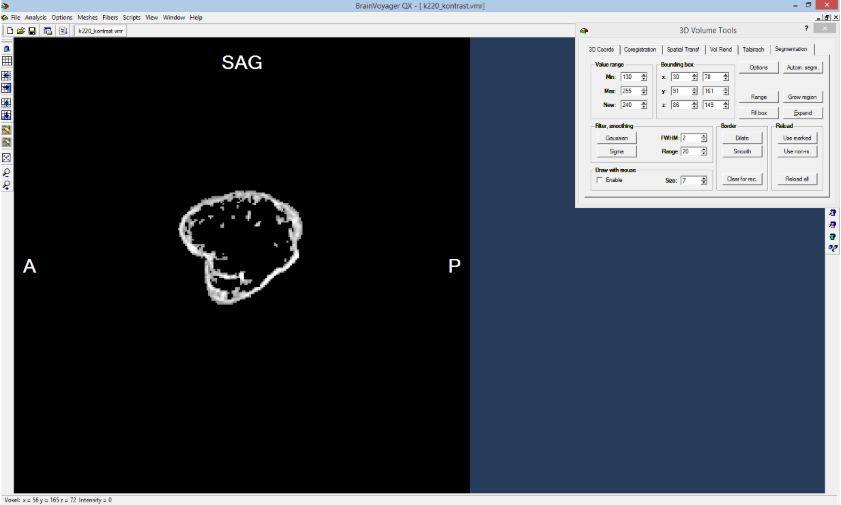


1. The voxels with non-enhancing tissue enclosed by the contrast enhancement were then segmented manually.


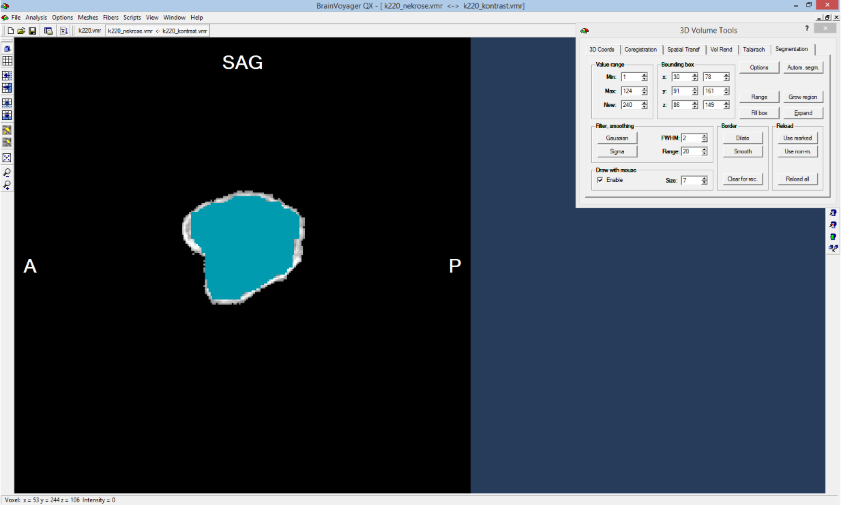

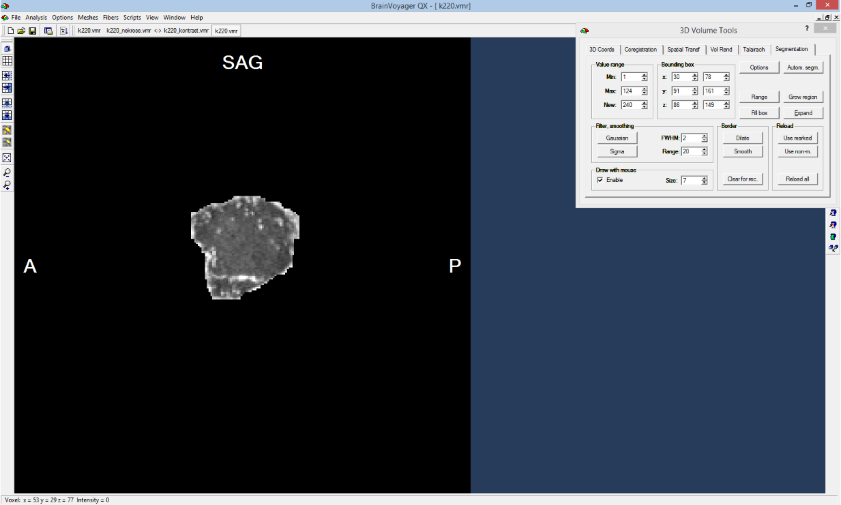


1. The contrast-label was used to remove all overlapping voxels from the necrosis-compartment.
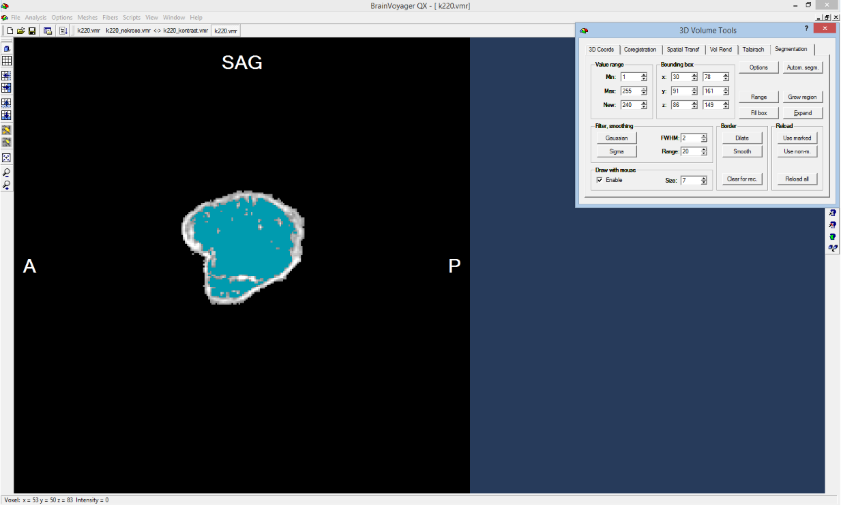

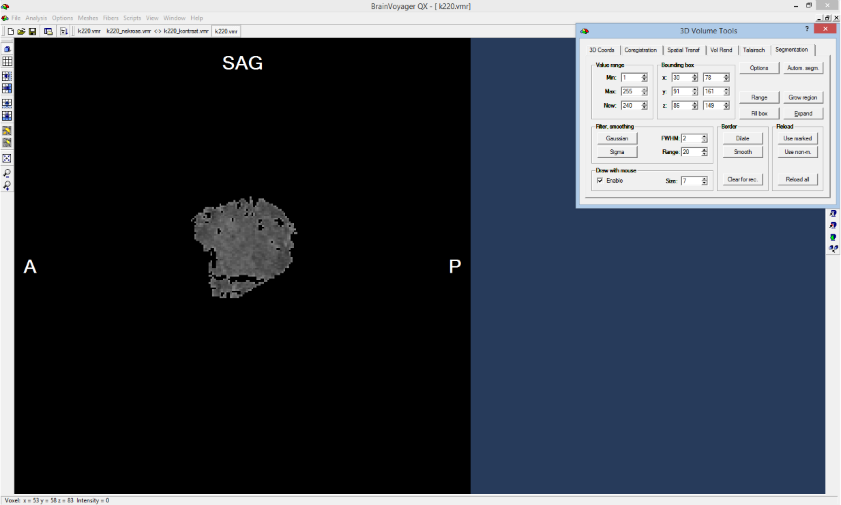

2. The number of segmented voxels was found using the Options>count voxels function, and the volume of the tumor was then calculated from the voxel-volume.

## Segmentation workflow in 3D Slicer

In 3D Slicer, a competitive region-based segmentation module called “GrowCut”, located in the editor module, was used.

1. Images were loaded in dicom format using the dicom browser.


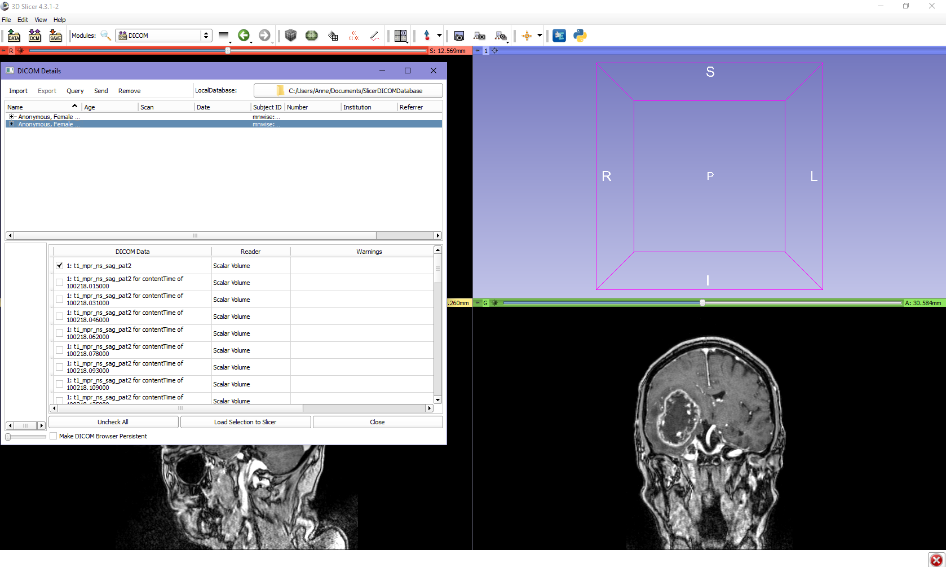


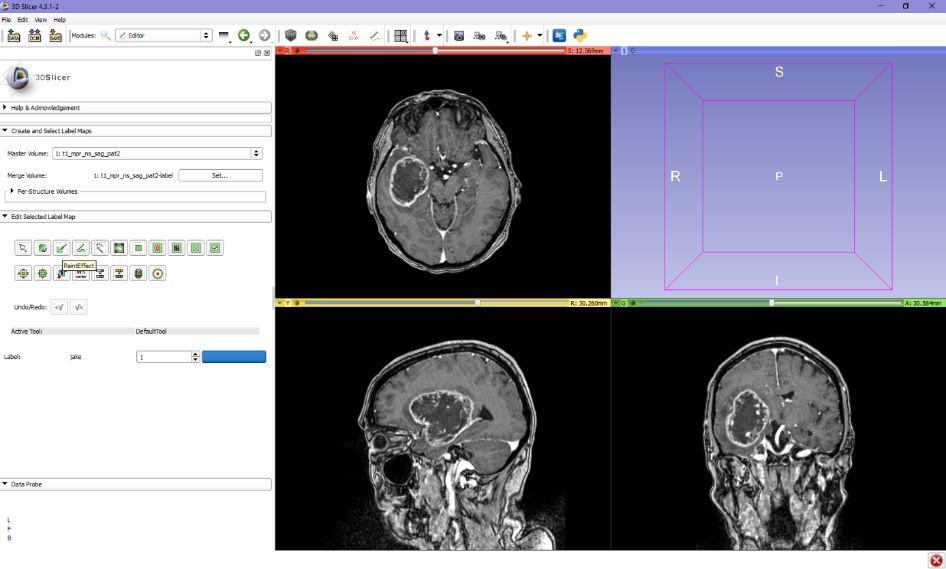


1. Using the “paint-effect” in the editor module (delineated in red), very rough outlines of the tumor were drawn in several planes in each directions using one label color.


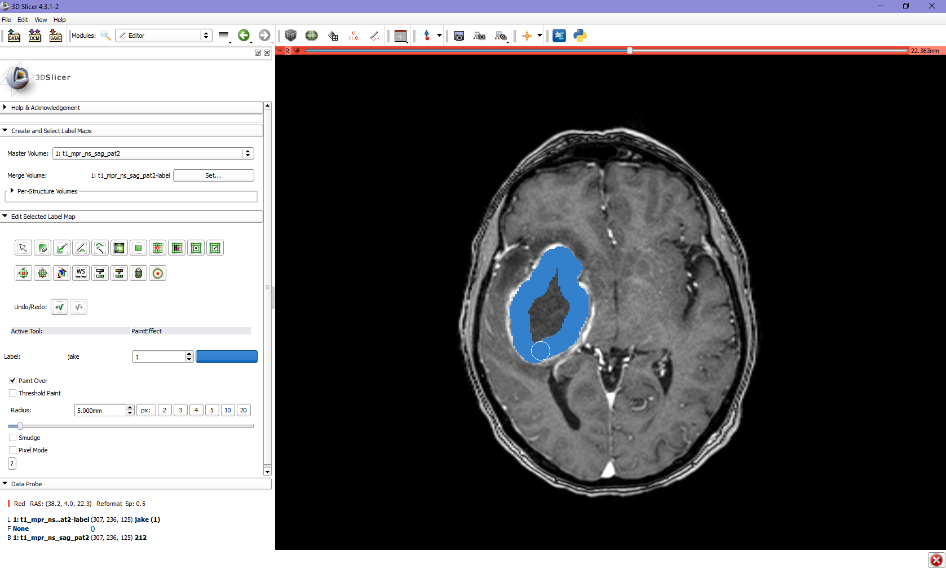


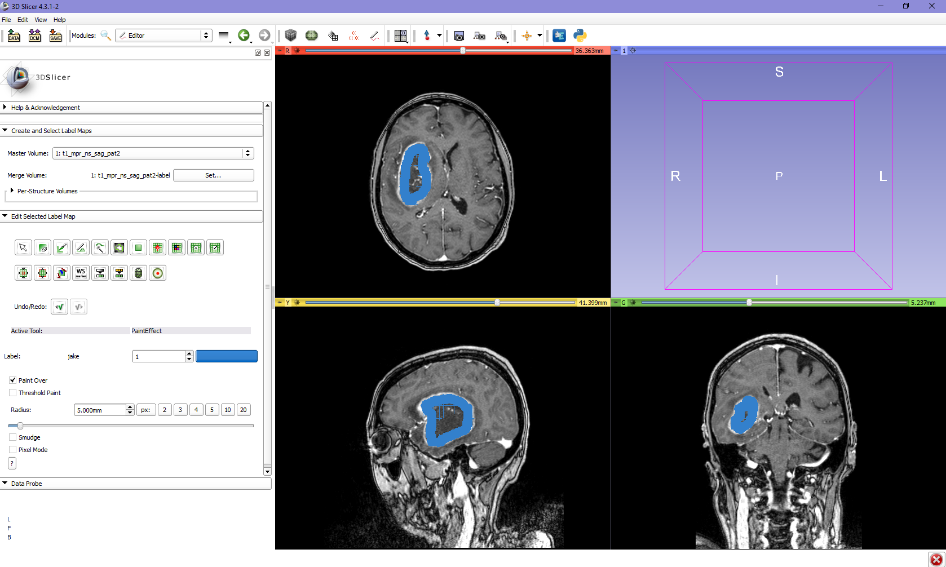


1. Then, a second label color was used to draw the outer borders of the tumor, to define “not-tumor” to the algorithm.


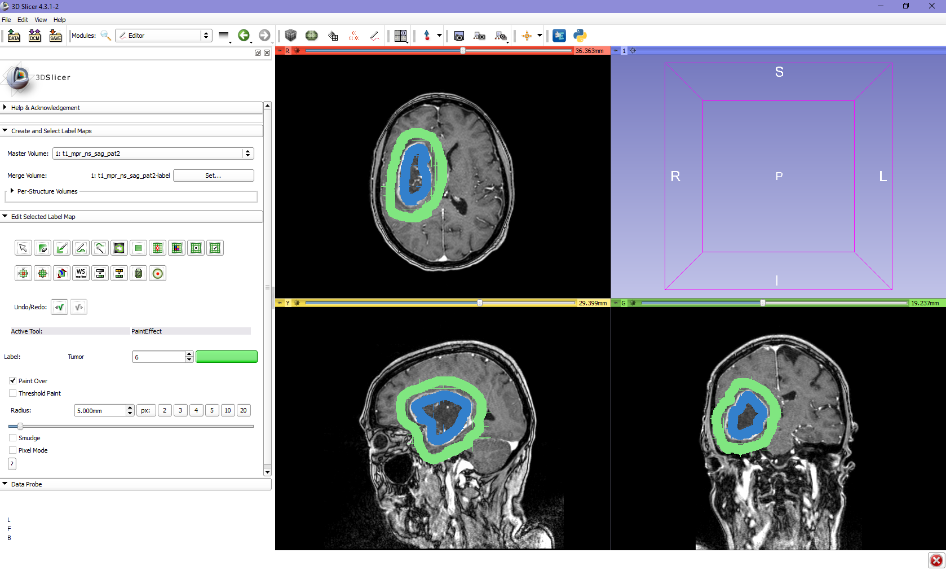


1. Using the tumor label color as the input, the grow cut algorithm (delineated in red) was run. This produces a complete, but rough segmentation label of the tumor.


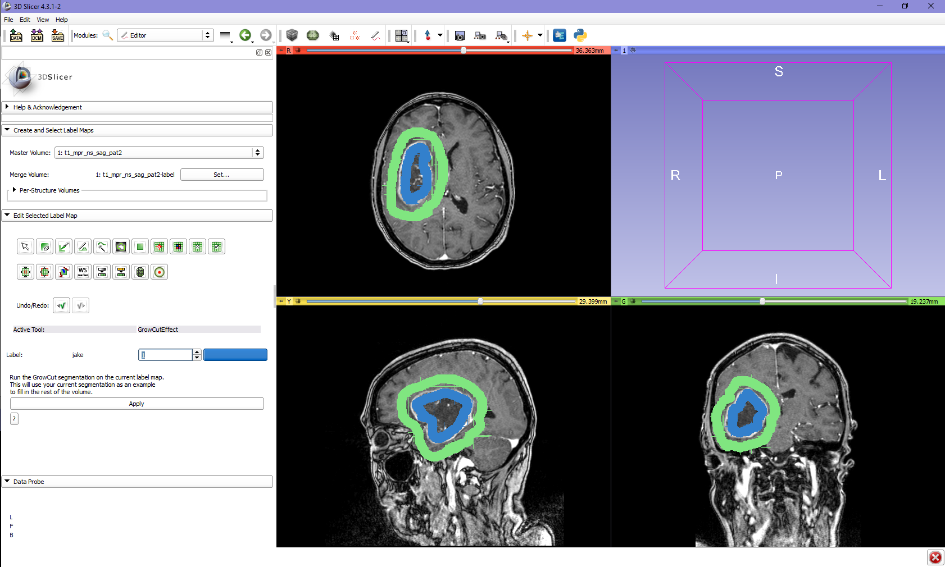


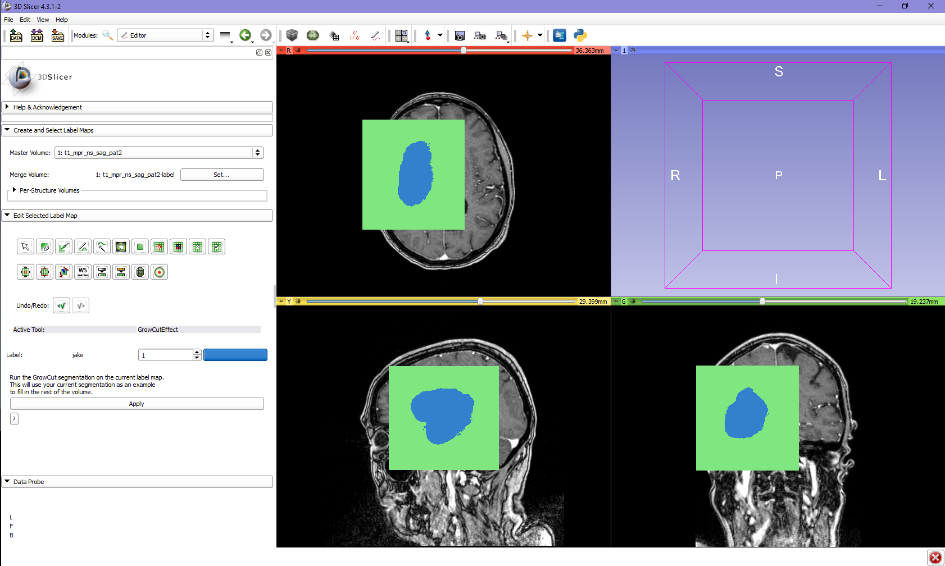


1. The “non-tumor” label was removed using the “change label” function (delineated in red)


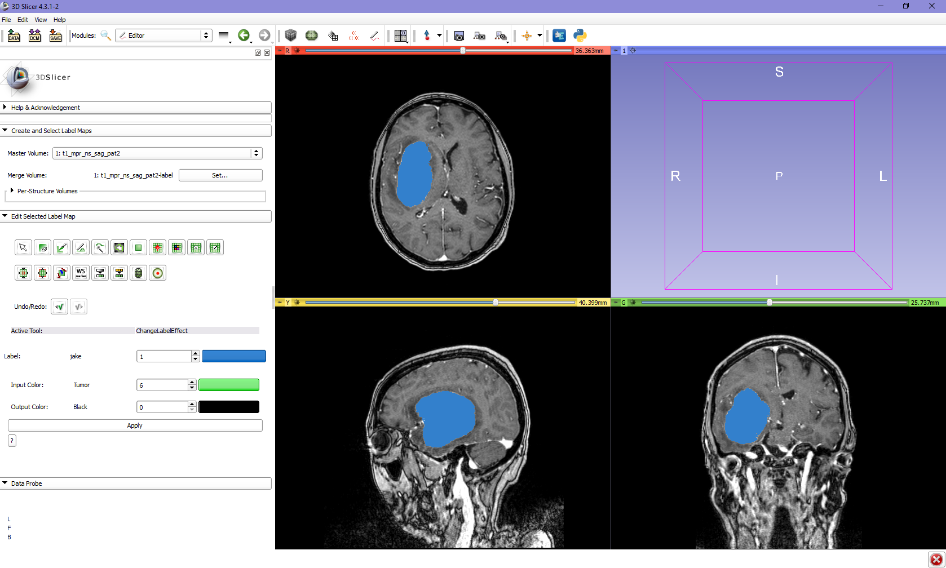


1. This label was then smoothened by using the “remove islands” (a) effect, and the “erode” (b) and “dilate” (c) functions in the editor module.
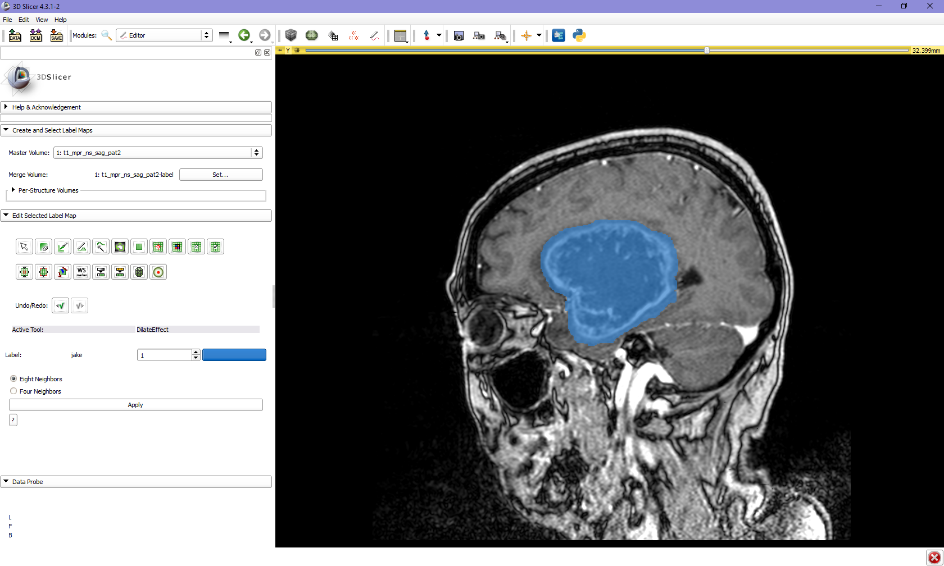

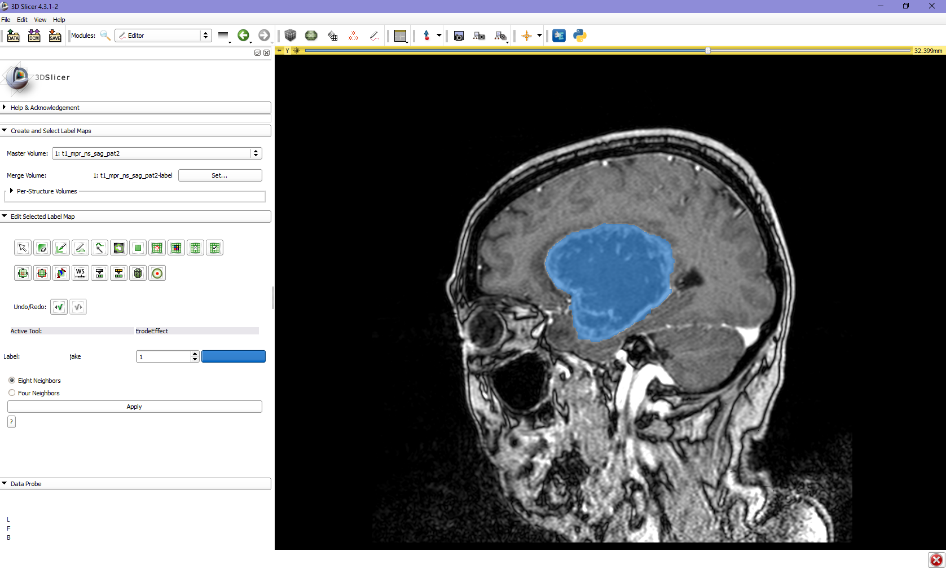


**c**

**b**

**a**

1. If necessary manually corrected using the “paint effect” function.


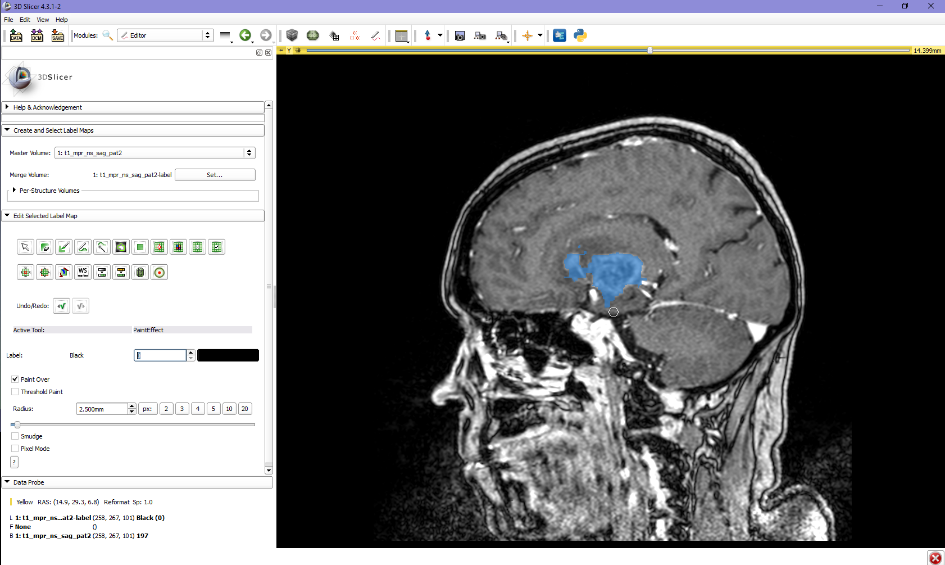


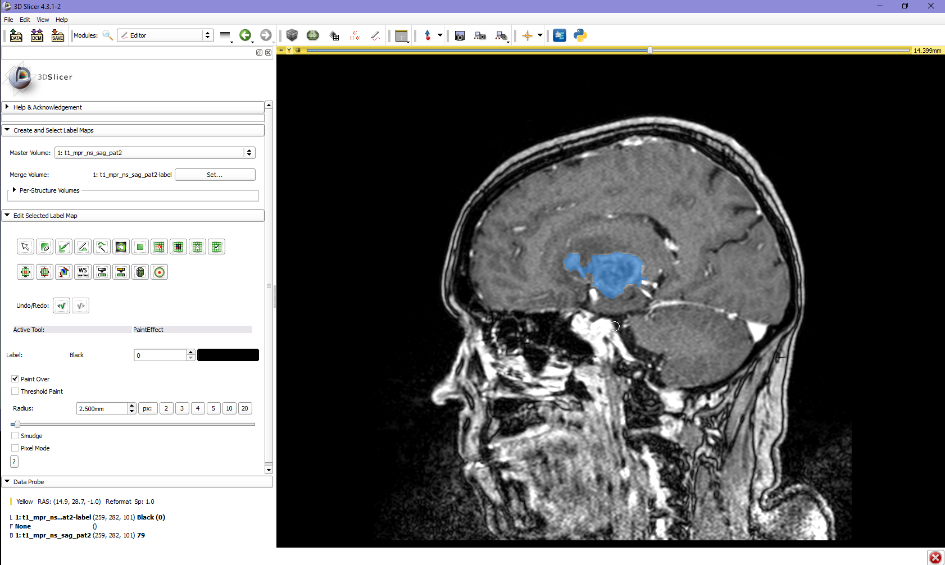


1. The label statistics module was used to find the volume of the tumor


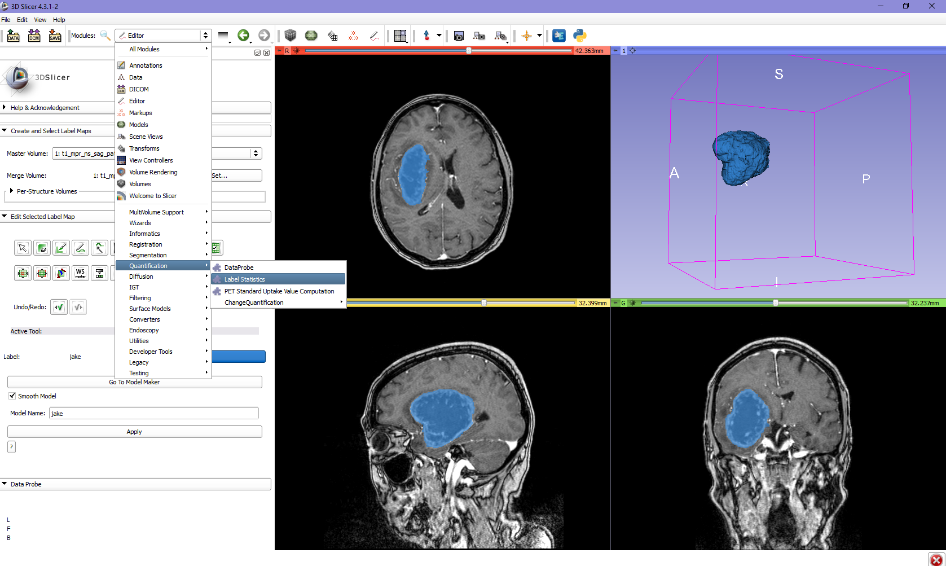


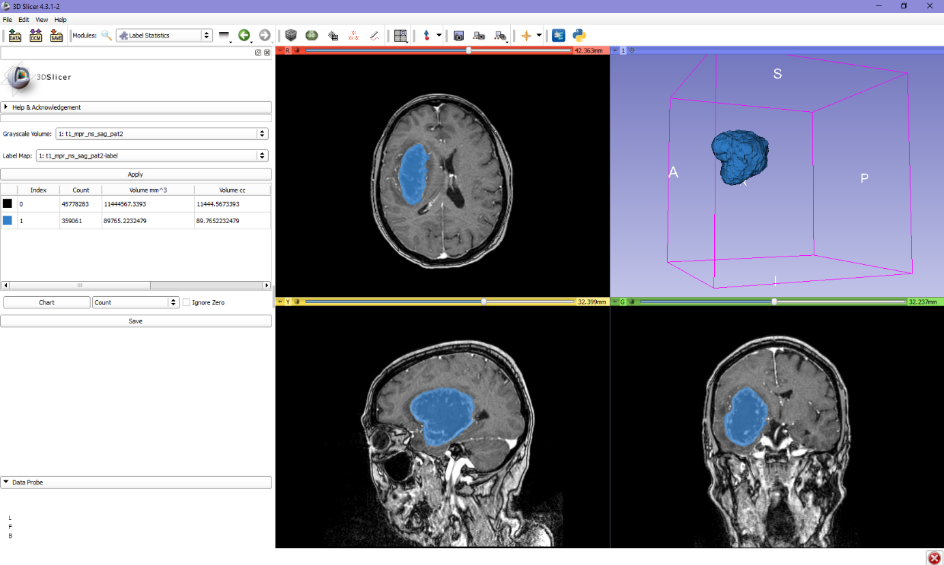


## Segmentation workflow in ITK Snap

Using the Active Contour Segmentation Mode with region competition, different growing points in the contrast-enhancing tumor parts were selected to “grow” the algorithm. The central non-enhancing tumor tissue often had to be manually added to the segmentation result.

1. Dicom images were loaded using the Open image function
2. Image contrast was adjusted using the auto-adjust option


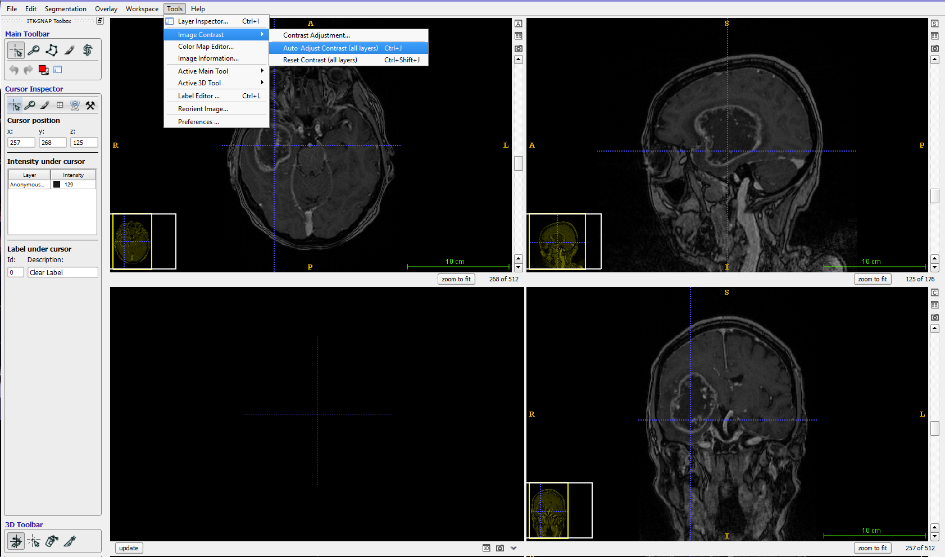


1. The active countour segmentation mode was used for the segmentation


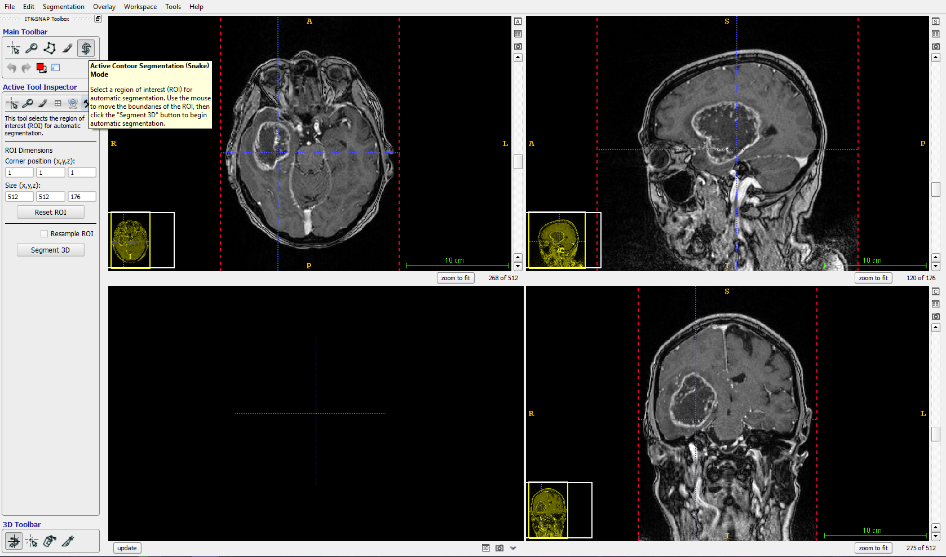


1. A ROI for the segmentation was manually adjusted


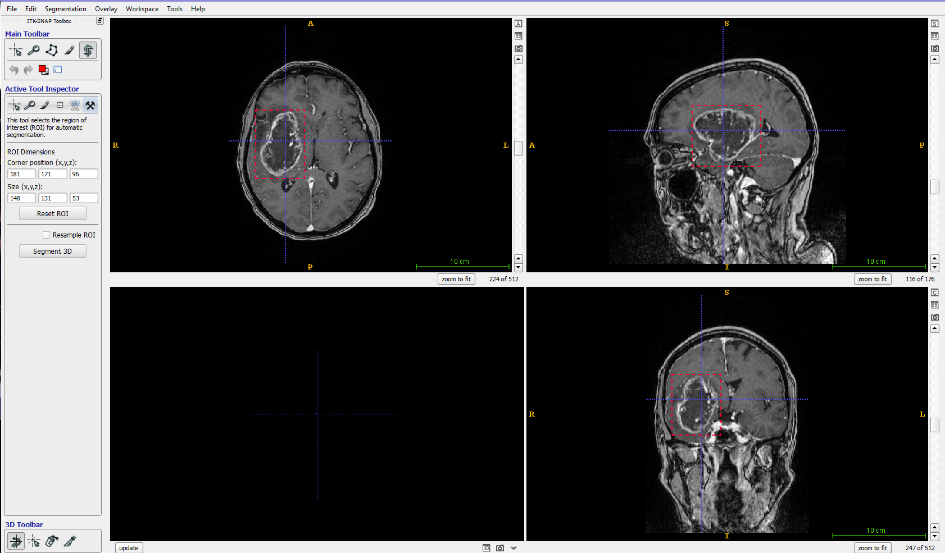


1. There are two options for the behavior of the active contour, either region growing or edge detection. We used region growing, which seemed to require less manual adjusting for glioblastomas.


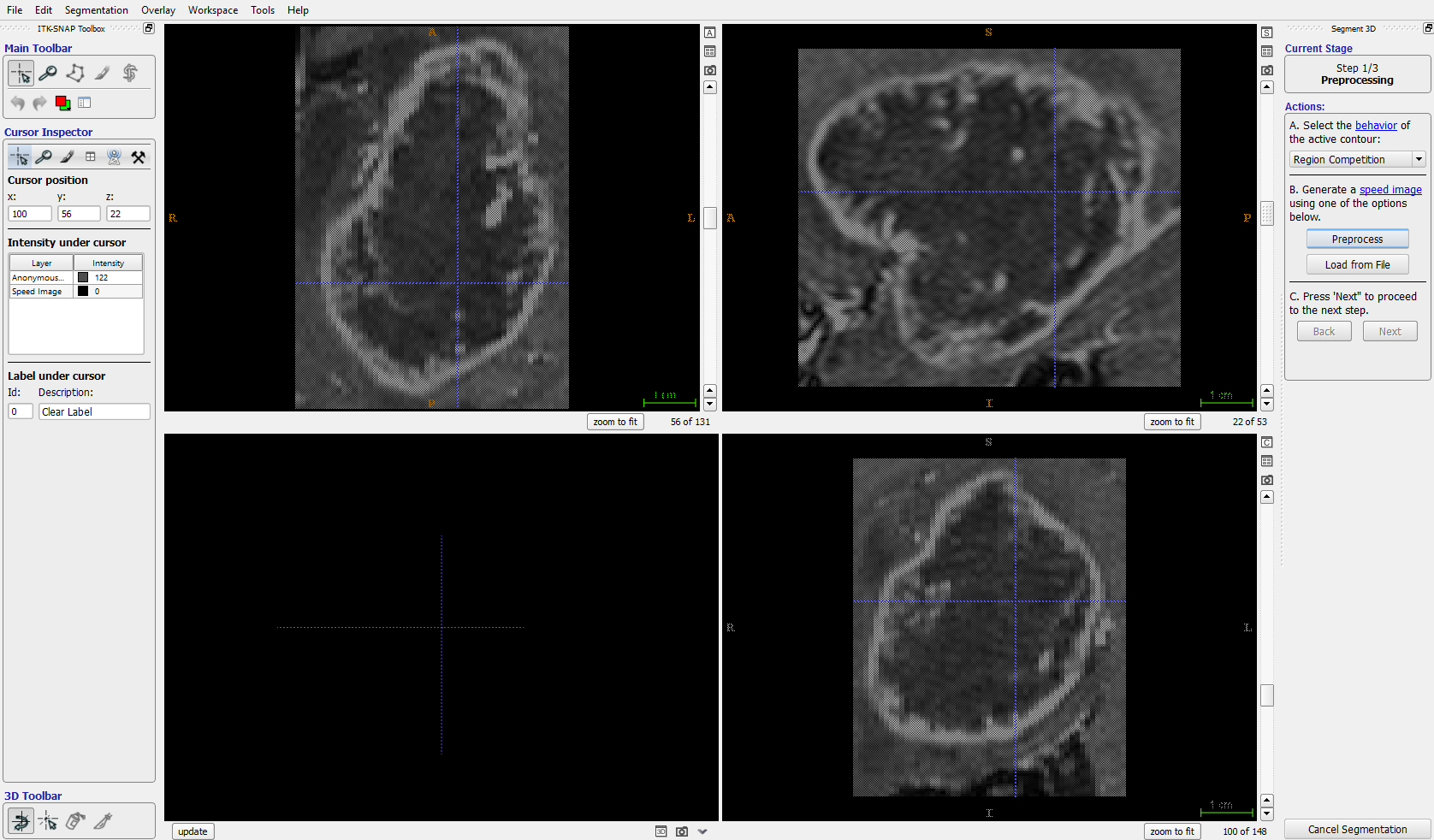


1. The first step of the segmentation was preprocessing, using a thresholding which was adjusted visually to the best result. This meant that the contrast enhancing part of the tumor and the necrosis had to be segmented separately.
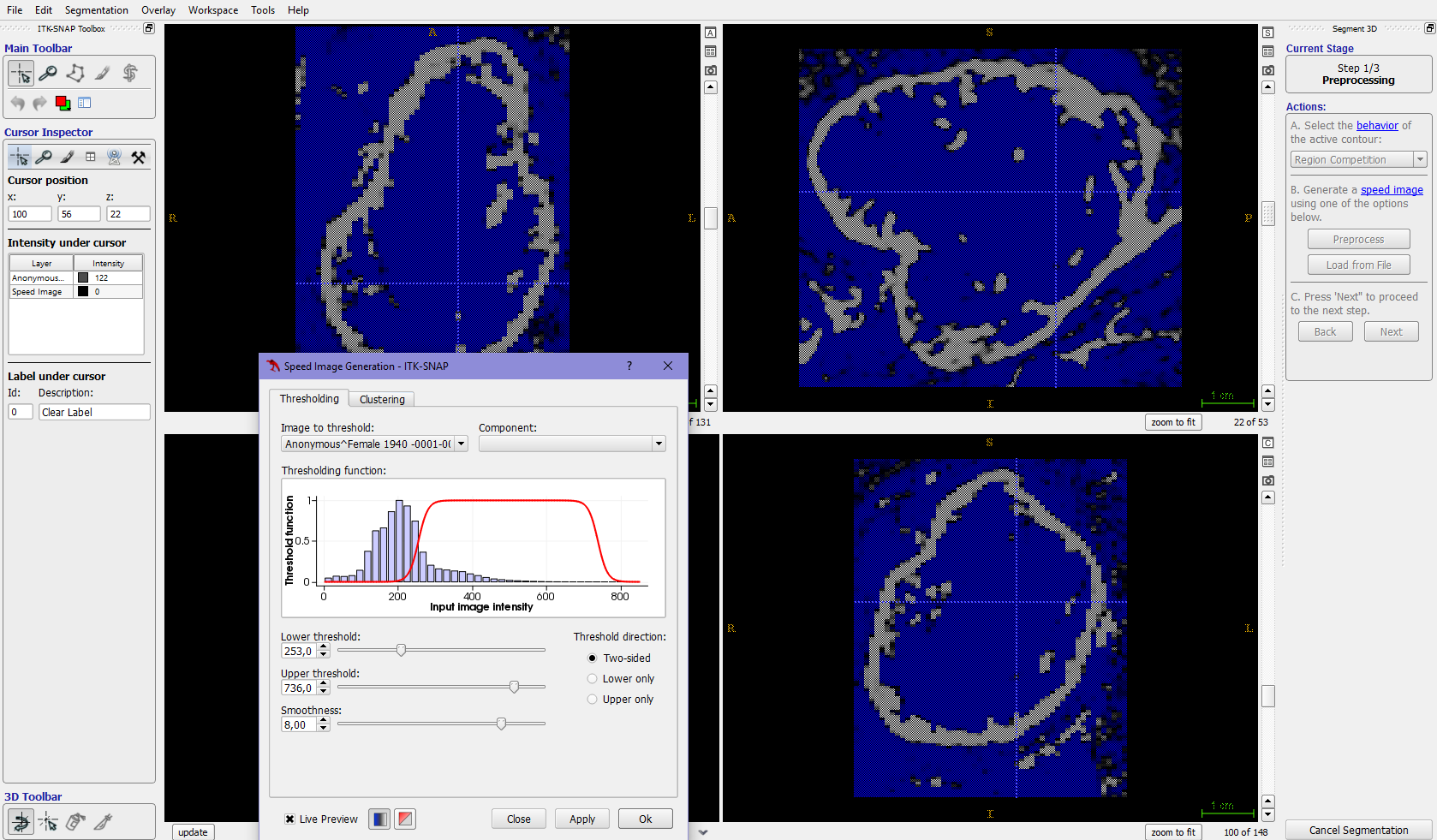

2. Then seeds, in the shape of three-dimensional bubbles, were placed on the resulting “speed map”


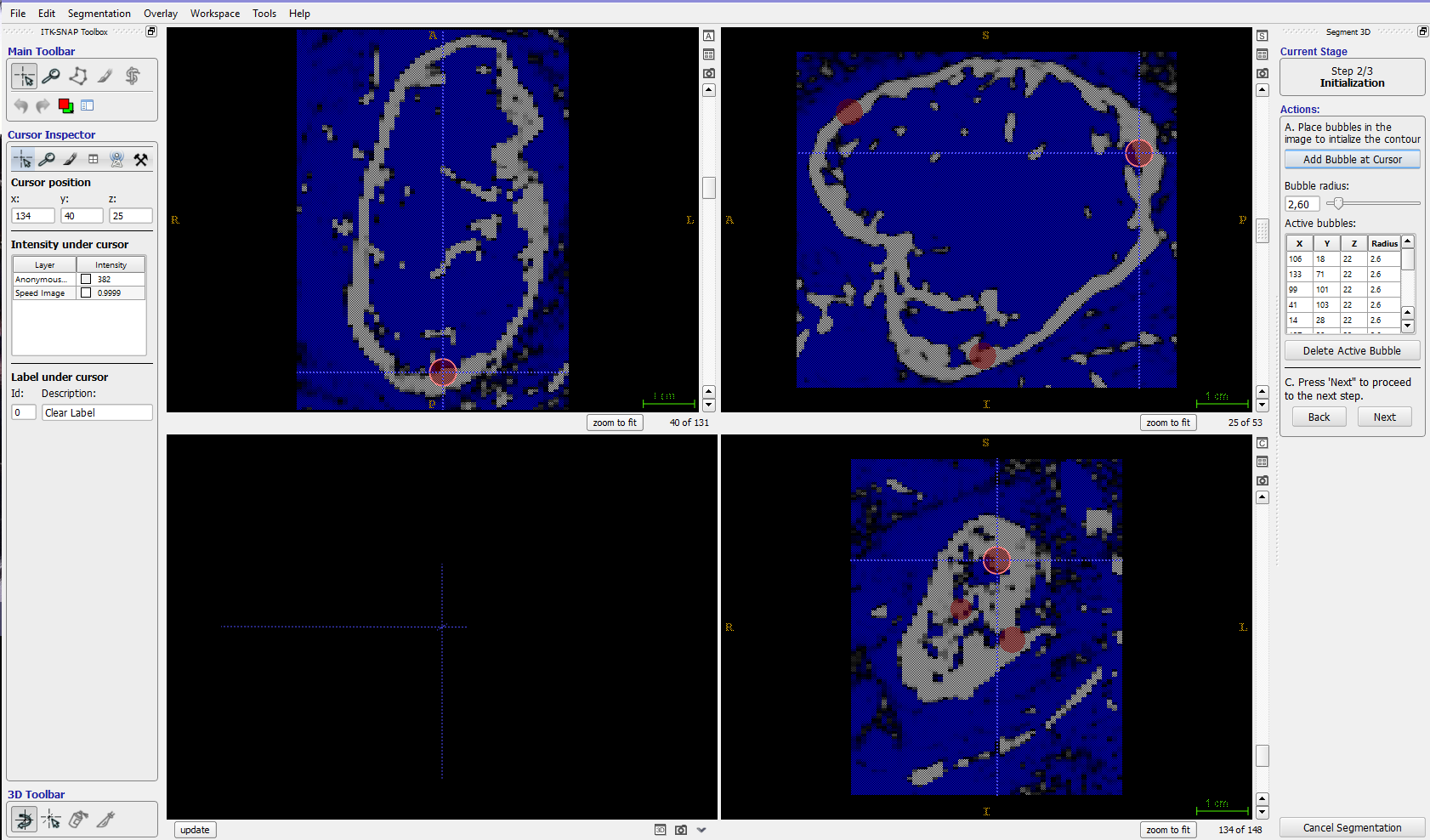


1. The next step was adjusting the parameters of the algorithm, a region growing force and a smoothing force. These were mainly adjusted if the segmentation result was unsatisfactory.
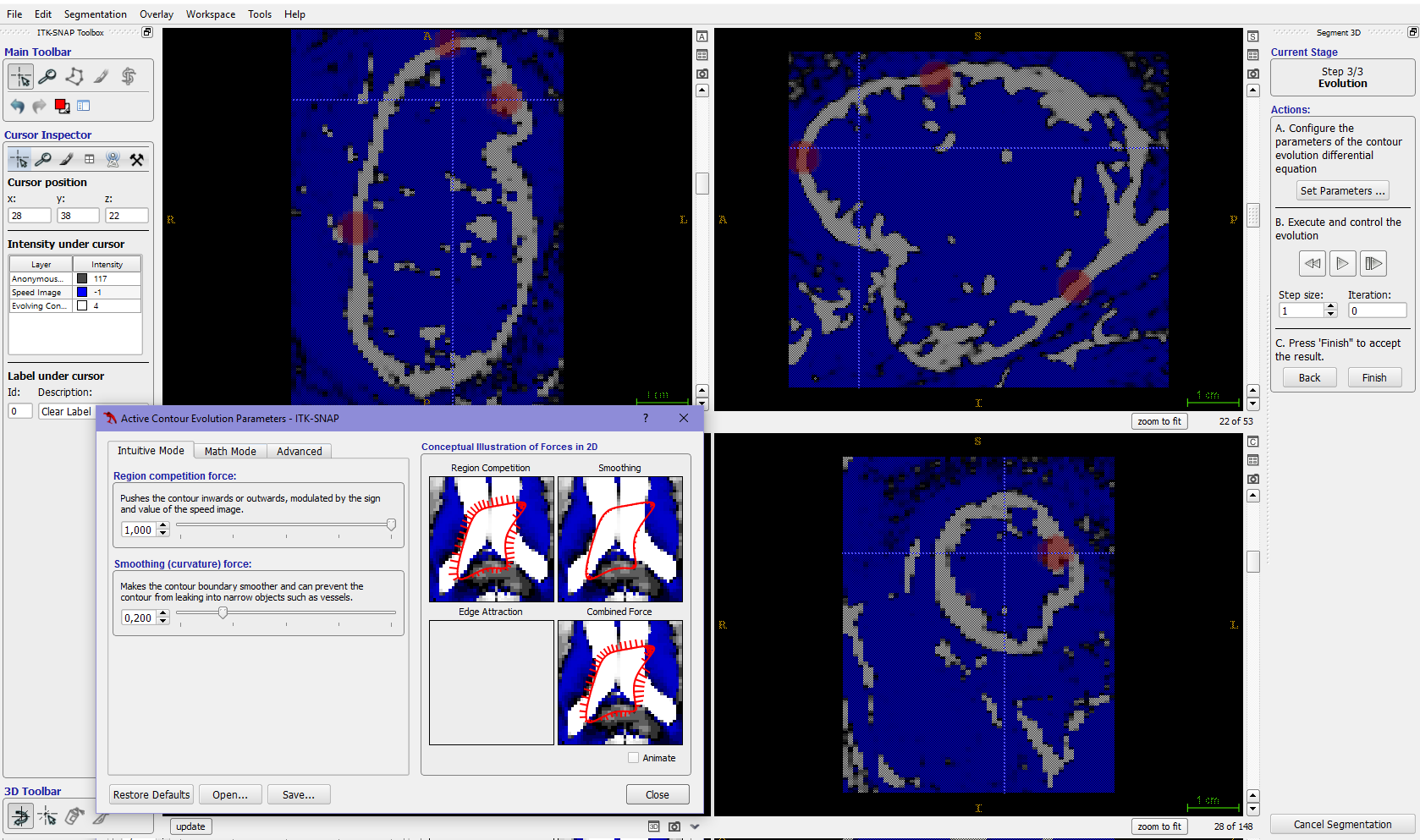

2. Then the algorithm was started, and the evolving estimate was graphically displayed as a color label. We stopped the algorithm when there was no further algorithm growth for approximately 5 seconds, or when the estimated tumor borders leaked outside the contrast-enhancing tumor.
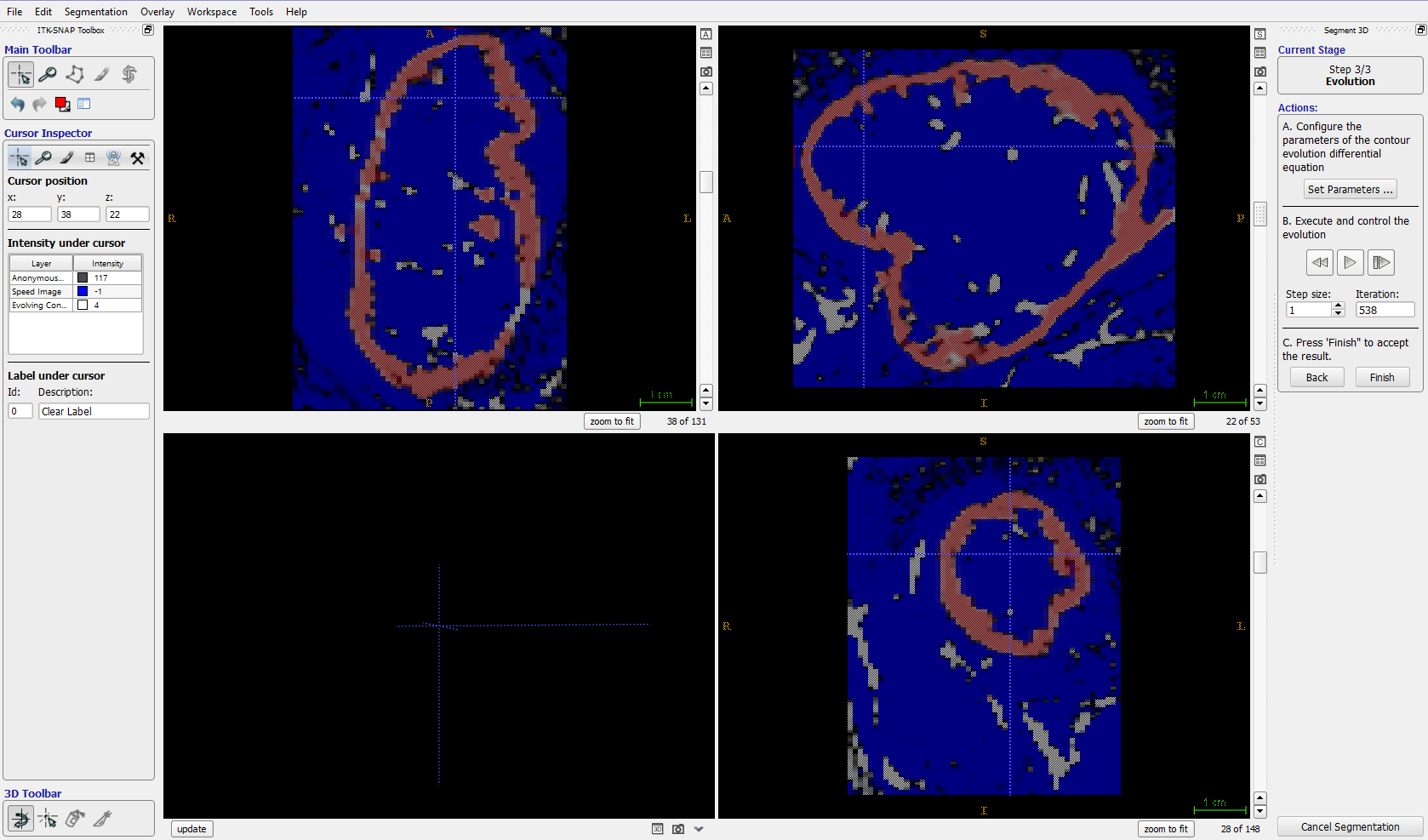

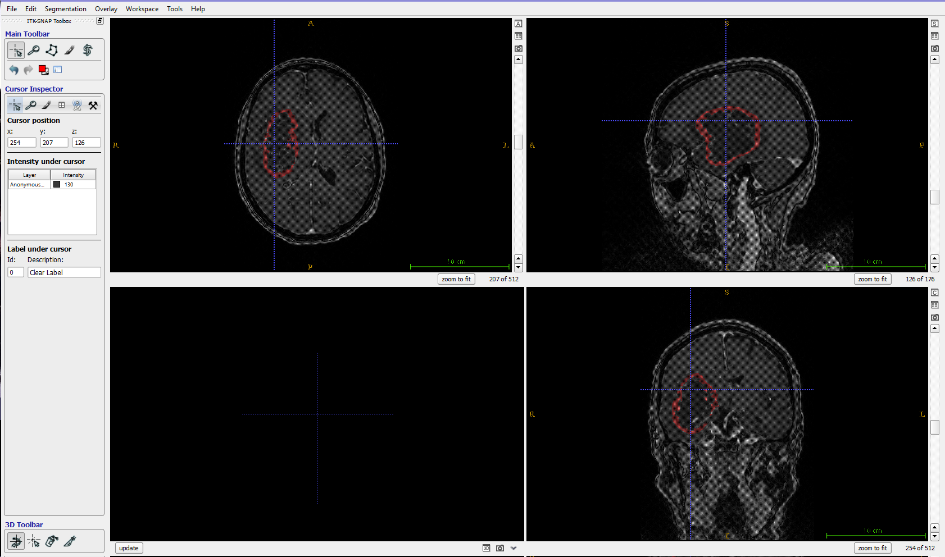

3. For small tumors, the central non-enhancing part of the tumor was manually added to the segmentation. For larger tumors, the steps above were repeated with a new label.
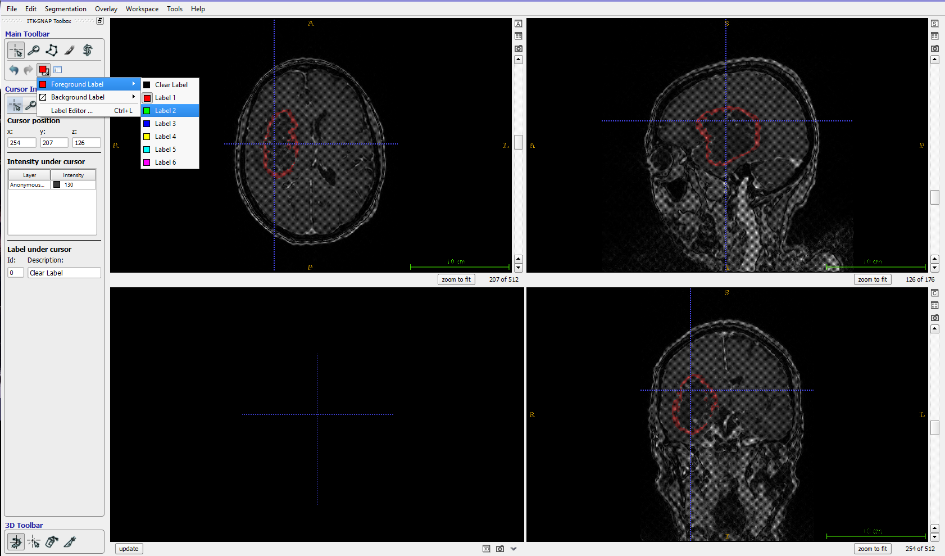

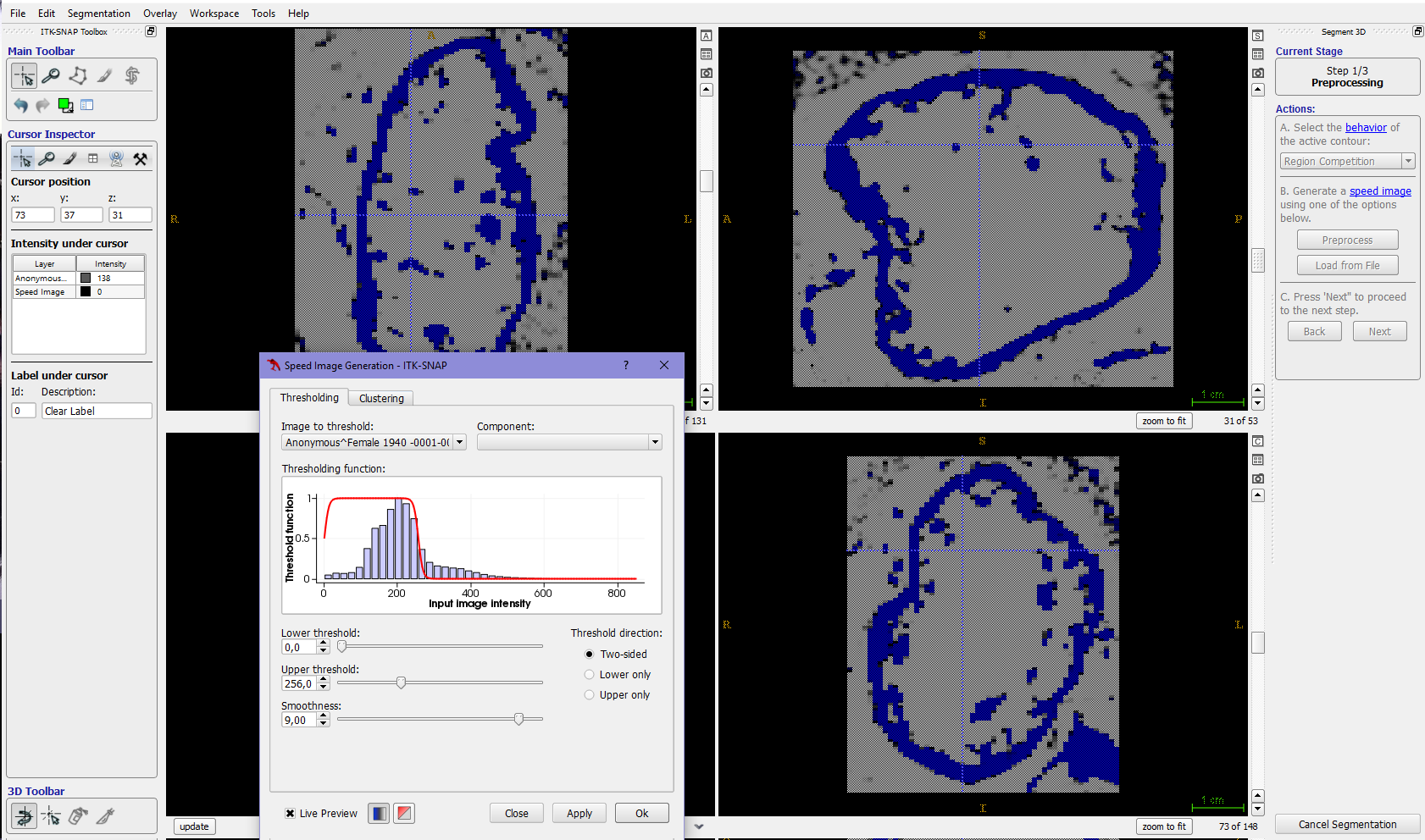

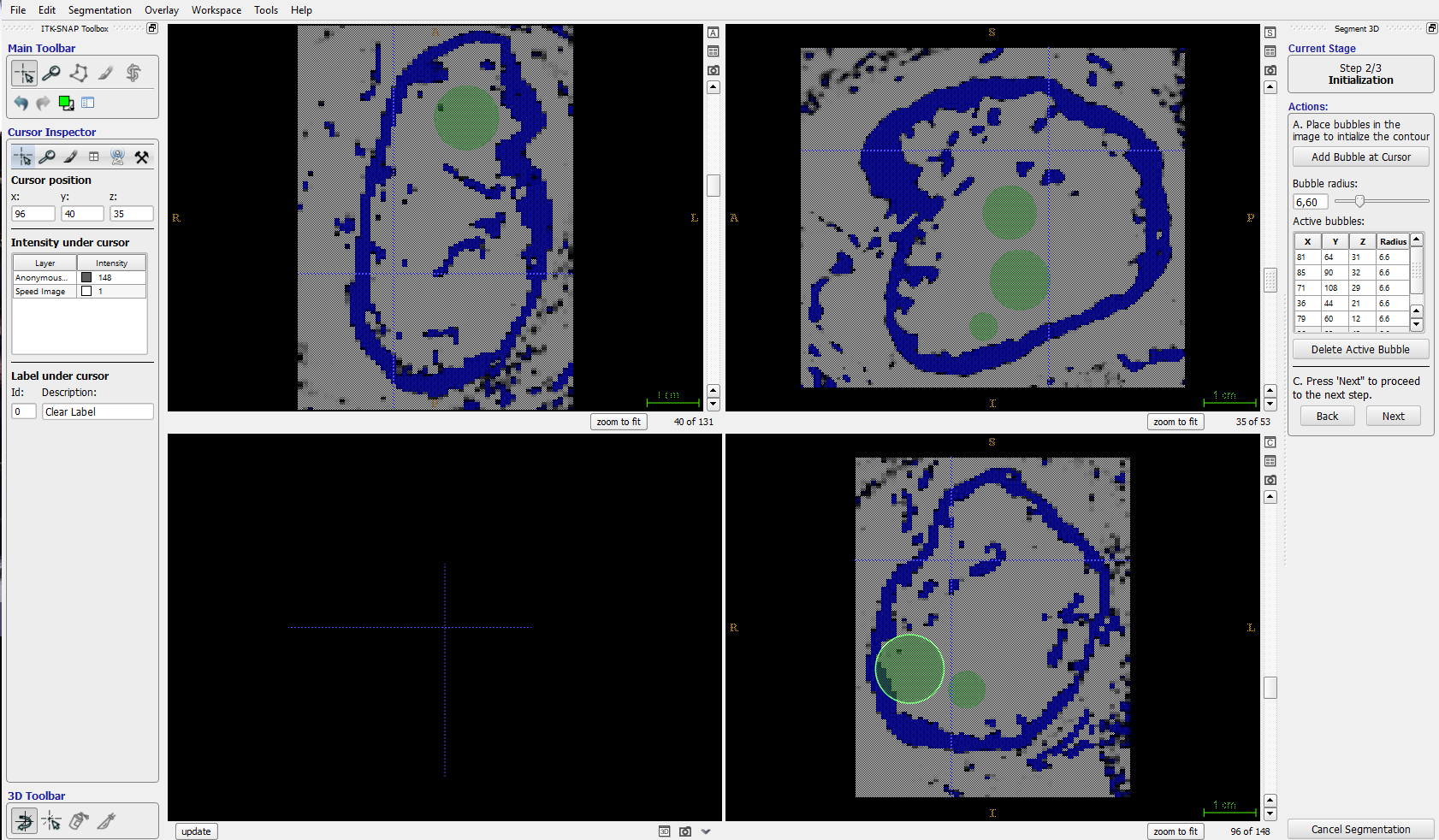

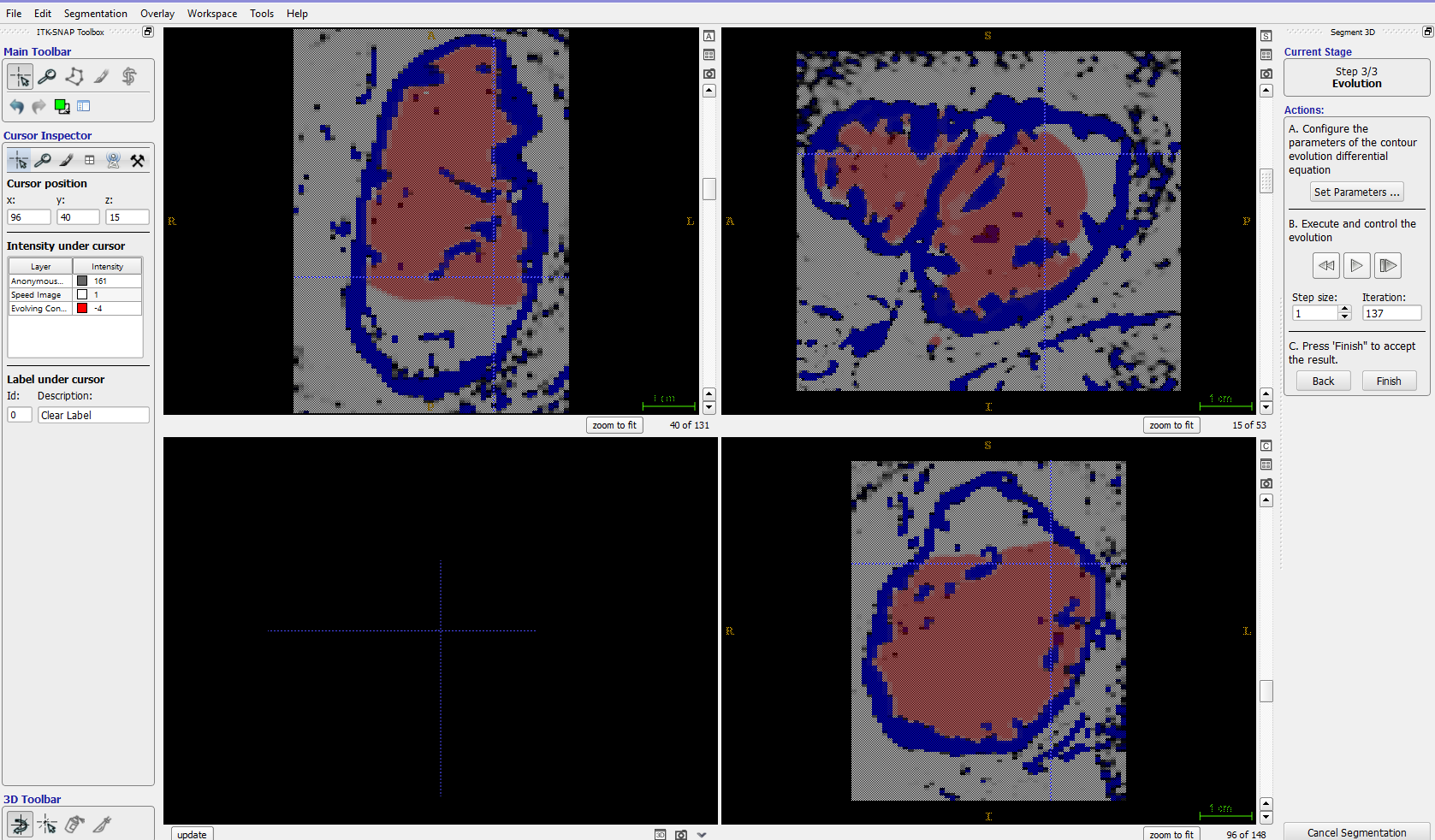

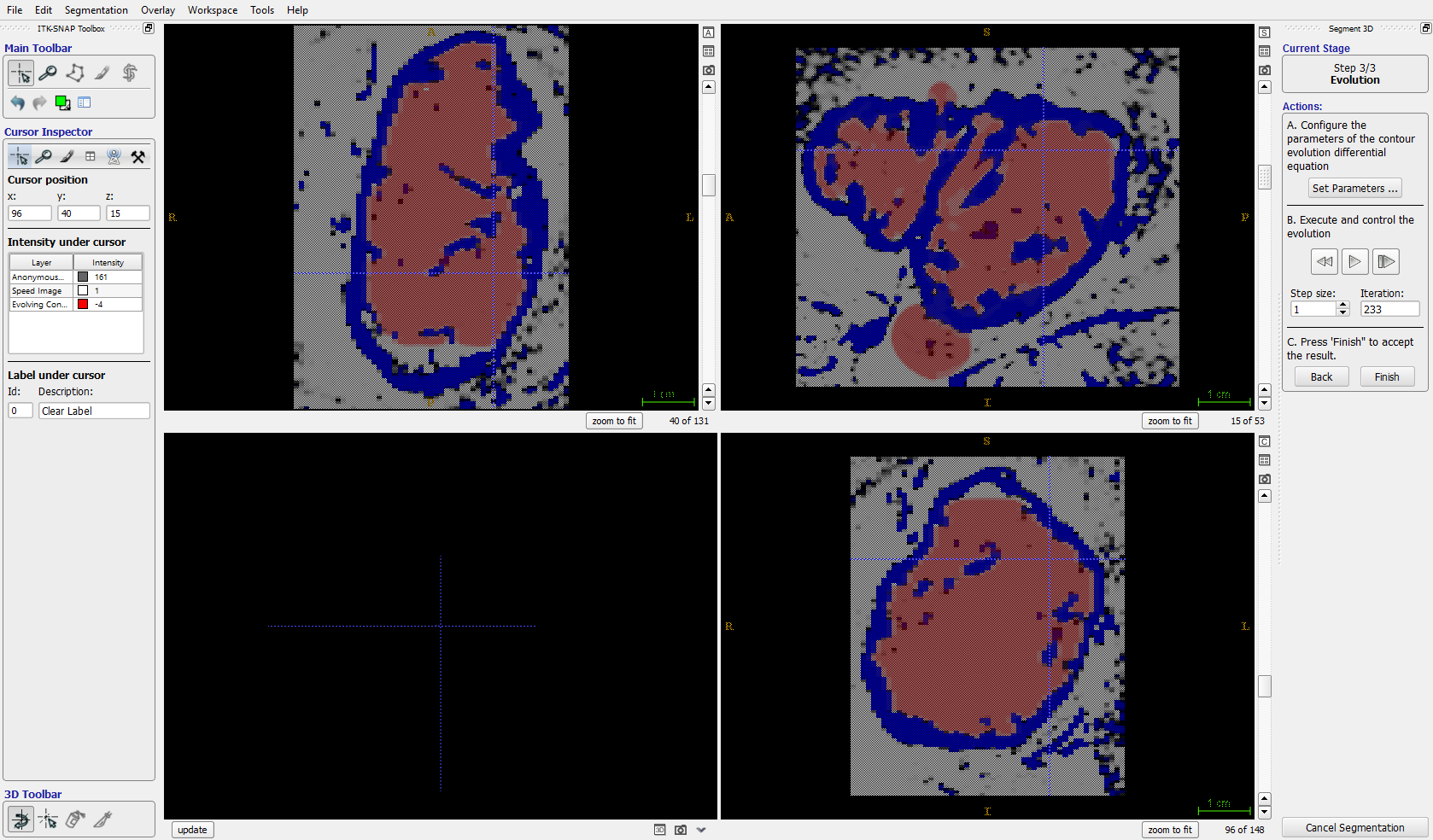

4. Then the results of both segmentations were manually corrected using the paint brush, and the non-enhancing tumor label was changes to the same color as the contrast enhancing compartment.
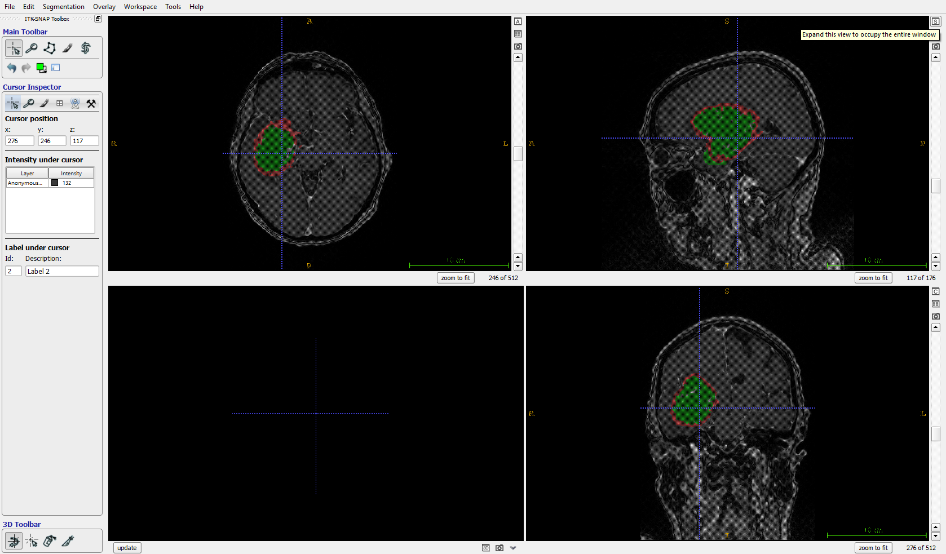

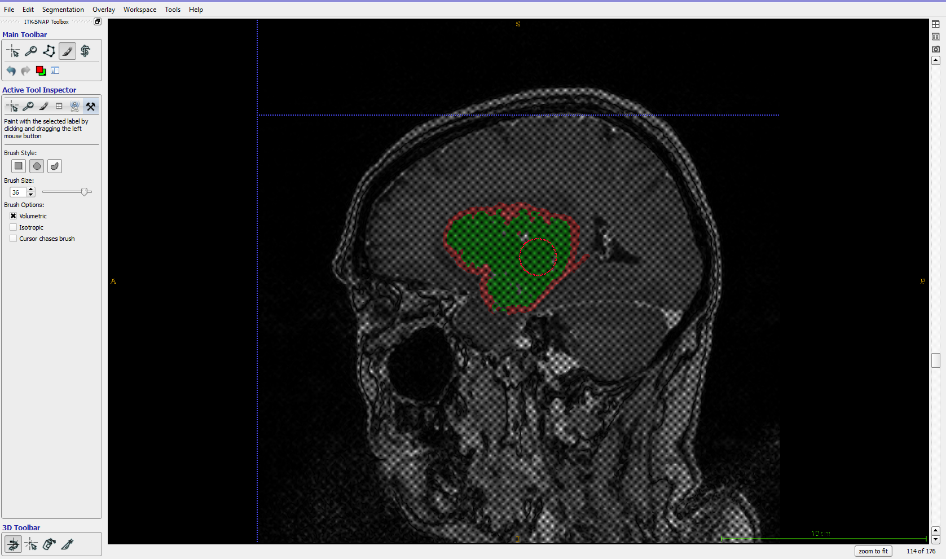


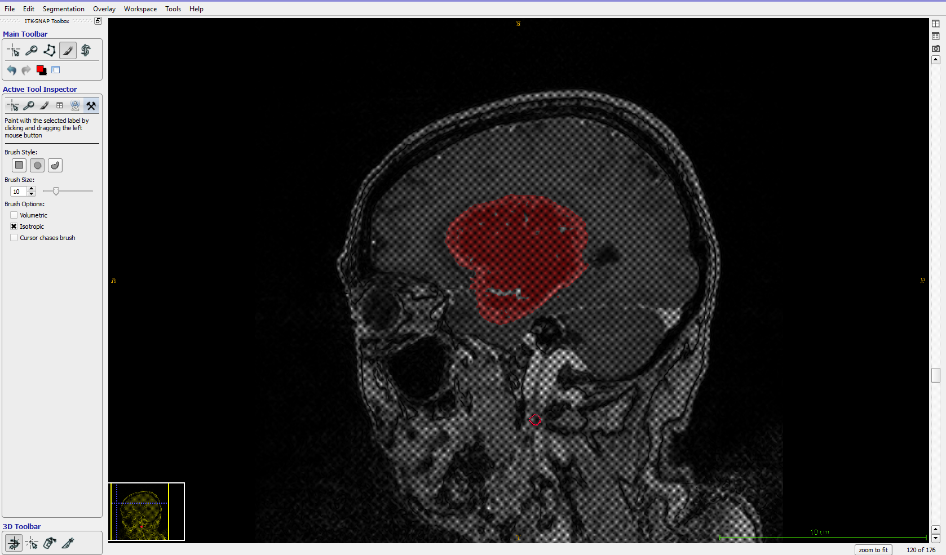

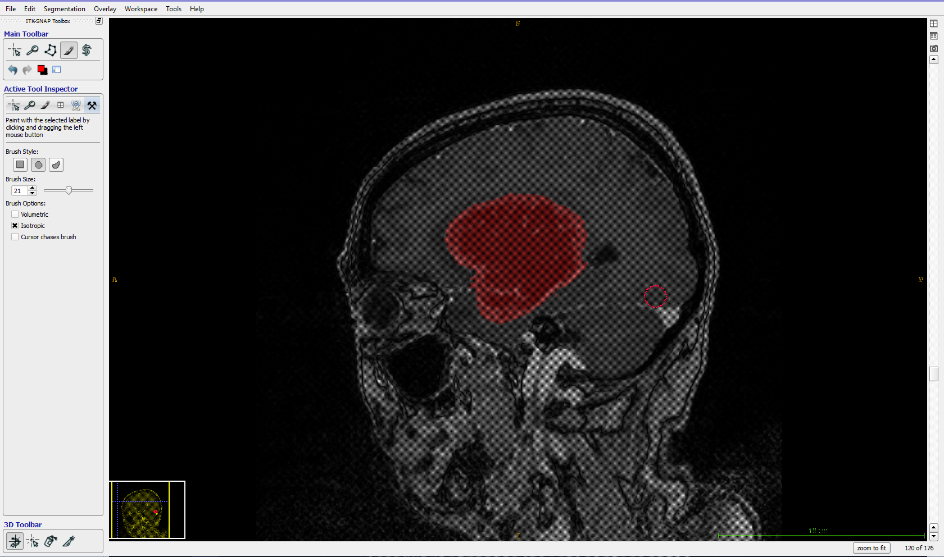


1. When the segmentation had been manually corrected, the segmentation volume was saved, and the tumor volume was found using the Volumes and statistics option.


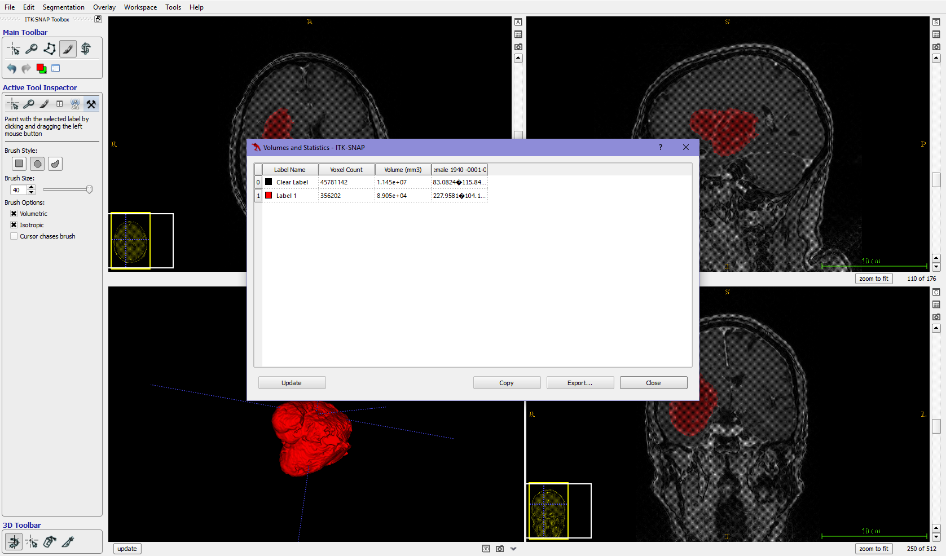

Supplement: S1 Appendix — (DOCX) [file pone.0164891.s001.docx]
