# Supplementary material for: Glioblastoma Segmentation: Comparison of Three Different Software Packages
Source: PLoS One. 2016 Oct 25;11(10):e0164891. doi: 10.1371/journal.pone.0164891 (PMC5079567; doi:10.1371/journal.pone.0164891)
Supplement: S1 Table — (DOC) [file pone.0164891.s002.doc]

| **Case** | **Scanner** | **Magnetic field strength (Tesla)** | **Slice thickness (mm)** | **Slice Spacing (mm)** | **Voxel area (mm^2^)** | **Image Resolution** | **Slice orientation** | **TR** | **TE** | **Flip angle** | **TI** | **Contrast bolus** |
| --- | --- | --- | --- | --- | --- | --- | --- | --- | --- | --- | --- | --- |
| **211** | Siemens Skyra | 3 | 1 | 0 | 1.000*1.000 | 256*256 | Sag | 1900 | 3.16 | 9 | 900 | 18 |
| **212** | GE Signa HDxt | 1.5 | 1 | 0 | 1.000*1.000 | 256*256 | Cor | 9.432 | 3.608 | 13 | 450 | ? |
| **214** | Siemens Skyra | 3 | 1 | 0 | 1.000*1.000 | 256*256 | Sag | 2000 | 2.96 | 8 | 1100 | 18 |
| **215** | Siemens Skyra | 3 | 1 | 0 | 1.000*1.000 | 256*256 | Sag | 2000 | 2.96 | 8 | 1100 | 15 |
| **216** | Siemens Avanto | 1.5 | 1.15 | 0 | 0.500*0.500^a^ | 512*496 | Sag | 2500 | 2.3 | 7 | 1000 | 20 |
| **217** | Siemens Avanto | 1.5 | 1 | 0 | 0.500*0.500^a^ | 512*496 | Sag | 2500 | 2.3 | 7 | 1000 | 19 |
| **218** | Siemens Skyra | 3 | 1 | 0 | 1.000*1.000 | 256*256 | Sag | 2000 | 2.96 | 8 | 1100 | 10 |
| **219** | Siemens Skyra | 3 | 1 | 0 | 1.000*1.000 | 256*256 | Sag | 2000 | 2.96 | 8 | 1100 | 16 |
| **220** | Siemens Avanto | 1.5 | 1 | 0 | 0.500*0.500^a^ | 512*512 | Sag | 2300 | 2.53 | 15 | 1100 | 15 |
| **221** | Siemens Skyra | 3 | 1 | 0 | 1.000*1.000 | 256*256 | Sag | 2000 | 2.96 | 8 | 1100 | 14 |
| **223** | Siemens Skyra | 3 | 1 | 0 | 1.000*1.000 | 256*256 | Sag | 2000 | 2.96 | 8 | 1100 | 20 |
| **224** | Siemens Skyra | 3 | 1 | 0 | 1.000*1.000 | 256*256 | Sag | 2000 | 2.96 | 8 | 1100 | 14 |
| **226** | Siemens Avanto | 1.5 | 1.05 | 0 | 0.500*0.500^a^ | 512*512 | Sag | 2500 | 2.3 | 7 | 1000 | 18 |
| **228** | Siemens Avanto | 1.5 | 1.1 | 0 | 0.500*0.500^a^ | 512*512 | Sag | 2700 | 2.25 | 7 | 1000 | 20 |
| **229** | Siemens Avanto | 1.5 | 1 | 0 | 0.500*0.500^a^ | 512*496 | Sag | 2500 | 2.3 | 7 | 1000 | 18 |
| **230** | Siemens Avanto | 1.5 | 1.1 | 0 | 0.500*0.500^a^ | 512*512 | Sag | 2500 | 2.3 | 7 | 1000 | 20 |
| **231** | Siemens Skyra | 3 | 1 | 0 | 1.000*1.000 | 256*256 | Sag | 2000 | 2.98 | 8 | 1100 | 20 |
| **232** | Siemens Avanto | 1.5 | 1 | 0 | 0.500*0.500^a^ | 512*496 | Sag | 2500 | 2.3 | 7 | 1000 | 16 |
